# The Growth Mindset of Beauty Promotes Risk-Taking Propensity and Behavior

**DOI:** 10.1177/01461672251327605

**Published:** 2025-04-02

**Authors:** Natalie T. Faust, Iris W. Hung

**Affiliations:** 1 NOVA School of Business and Economics, Carcavelos, Portugal; 2 The Chinese University of Hong Kong, Shenzhen, China

**Keywords:** implicit theories, beauty, risk-taking, optimism

## Abstract

Beauty has pervasive implications for success in various domains of life. Given this broad and visible nature, whether and how a belief in the improvability of this important human attribute influences judgment and decision-making is largely unknown. We found that beauty implicit theories can produce strong cross-domain impact on risk-taking behavior. Using both hypothetical choices and real behaviors in one cross-country survey and nine experiments, including three supplementary studies (*N* = 4,015), we found that (a) incremental theorists, who believed that beauty is malleable and improvable, took greater risks than entity theorists, who believed that beauty is fixed, and (b) an incremental belief of beauty heightens a sense of optimism that one will achieve positive outcomes in various domains of life, which consequently promotes risk-seeking behavior. These findings demonstrate that domain-specific implicit theory (i.e. beauty in our case) can affect behavior beyond that domain (non-beauty related risk-taking).

Surely beauty matters. The impact of beauty on our lives is undoubtedly pervasive. Along with other human characteristics, beauty is an important quality through which individuals assess themselves and others. Equally ubiquitous is the concept of beautification. As of 2023, beauty is the third most popular category among global Instagram influencers, only after music and lifestyle (Statista, 2024a). The thriving beauty industry prompts people to care about their appearance and attend to various seemingly viable means of beautification. Beyond holding a more subjective view that beauty is “in the eyes of a suitable beholder,” people spend considerable effort to enhance their beauty. One means is via the use of cosmetics, with the global cosmetics market estimated to value at 758.4 billion U.S. dollars by 2025 (Statista, 2024b), even higher than the GDP of Belgium (689.36 billion U.S. dollars) which is ranked 23 of the major economies (Investopedia, 2024). Even during the COVID-19 pandemic, beauty products have been considered an essential commodity (CNBC, 2020). Being able to beautify the self has had “currency” probably since before at least 2,500 years ago when people started to debate about what beauty is (Feagin, 1995; Reber et al., 2004; Tatarkiewicz, 1970). Beauty signals success in life. It is positively correlated with social and professional skills (Langlois et al., 2000). In labor economics, physically attractive workers are considered to be more able by employers (Mobius & Rosenblat, 2006). In the present research, we ask several fundamental questions. Given the pervasive roles that beauty has in multiple domains of our life, do beliefs in the improvability and malleability of this human attribute influence decision making? If so, what are the nature of these effects? We attempt to answer these questions by examining the effect of implicit theories of beauty on risk-taking behavior.

These research questions are unique and important because of theoretical and practical reasons. First, understanding whether and how beliefs pertaining to beauty may play a role in behavior and decision-making is important given that individuals are bombarded by numerous media messages on the notion of beautifying the self on a daily basis. Second, human traits and attributes do not only make a direct impact on behavior and decision-making but also *indirectly* via implicit theories, which shape how people make judgment about the self and others. Surprisingly, how beliefs or mindsets about beauty, which is one of the most important human attributes, influence human behavior, is largely unknown. Third, most research in this area has mainly focused on the effect of implicit theories on how people process information (encoding, attention; Hong et al., 2009) and form judgment about the self and others *within* the same domain of interest (Dweck et al., 1993, 1995; Dweck & Leggett, 1988). Implicit theories may influence within-domain self-regulatory processes and affective, cognitive, and behavioral outcomes. Renowned researchers have urged for an increased attention to a follow-up research avenue, namely, how implicit theories influence cross-domain judgment and decisions (e.g. Molden & Dweck, 2006). This research attention is particularly germane to the beauty domain because of the broad nature of beauty (i.e. beauty is considered to be correlated with reproduction ability, financial success, and positive trait perceptions such as credibility). In the current research, we asked whether and how implicit theories of beauty may transcend the beauty domain and influence non-beauty related risk-taking behavior. Fourth, the current research provides robust evidence that uniquely distinguishes itself from previous studies.

In the remainder of this article, we first describe our theorizing of how implicit theories of beauty may be related to risk-taking. Next, we present a set of seven studies, and three supplementary studies in Supplemental Materials, which examined the causal influence of beauty implicit theories on risk-taking indirectly through felt optimism that positive outcomes would occur in many domains in life. Finally, we discuss the theoretical and practical implications of the current research.

## Implicit Theories of Beauty

Scholars (Chiu, Hong, et al., 1997; Dweck et al., 1995; Hong et al., 1997) have identified two types of implicit theories: an entity theory, which refers to the belief that human attributes and characteristics are fixed and cannot be changed, and an incremental theory which sees them as changeable and improvable with effort and able to be cultivated. Implicit theories have been shown to influence attitudes and behavior across various domains, such as intelligence (Hong et al., 1999; Nussbaum & Dweck, 2008), race (Tadmor et al., 2013), morality (Chiu, Dweck, et al., 1997), and sexuality (Maxwell et al., 2016). Numerous studies have demonstrated how implicit theories influence information processing and decision-making (Dweck et al., 1993, Kwon & Nayakankuppam, 2015; Mathur et al., 2012). For example, entity (incremental) theorists of intelligence tend to make trait (effort) attributions when evaluating failures of themselves and others (Dweck et al., 1993); entity (incremental) theorists of moral characters, meanwhile, tend to make stronger (weaker) trait-attributions for others’ recent behavior as well as more long-term behavioral predictions (Dweck et al., 1995).

Previous findings on implicit theories have primarily focused on trait-like attributes (e.g. intelligence, morality, personality, race, gender roles) and transient states (e.g. emotion, feelings of interest, shyness). Surprisingly, not much attention has been paid to beauty. There are two exceptions which have robustly confirmed in both the field and lab that beauty implicit theories exist (Burkley et al., 2014; Faust et al., 2024). Unlike most human attributes that have been studied (e.g. intelligence, morality, emotions, and passion), beauty is a human attribute that is immediately visible to the external environment. Its visibility may contribute to its immediate impact on perceptions in social and professional domains. Beautiful people are perceived to be capable and paid good salary in labor market (e.g. Mobius & Rosenblat, 2006). Intelligence and morality do not necessarily bring similar, immediate impact on professional success and social perceptions. A meta-analysis showed that a relatively larger proportion of physically attractive adults (68%) are high income earners, compared to that among physically unattractive adults (32%) (Langlois et al., 2000). Given that beauty has such a strong and direct effect on individuals’ decisions across a broad number of domains (reproductive, social, and professional), it is reasonable to assume that how people believe about beauty (i.e. implicit theories) may bring strong and unique impact on decisions related to risk-taking. Understanding how implicit theories of beauty influence decision-making and behavior is important because human attributes and traits may influence judgment and behavior both *directly* (e.g. via beauty) and *indirectly* (via implicit theories about beauty).

## Implicit Theories of Beauty, Optimism, and Risk-taking

The present research is focused on the indirect effect of beauty theories on individuals’ behavior. We conjecture that they provide distinctive effects on (a) general risk-taking behavior, and (b) this is because the broad and visible nature of beauty elevates a sense of optimism that positive outcomes would occur in various domains in life (Scheier & Carver, 1985; Scheier et al., 1994). Given the breadth of impact of beauty on multiple domains, it is reasonable to argue that implicit theories of beauty may produce cross-domain influences. We define cross-domain influences as the impact of beauty implicit theories that occur in seemingly unrelated domains in life. Previous research has provided some evidence for the relationship between beauty and risk-taking. For example, a study showed that young men tend to take more physical risk in the presence of an attractive female (Ronay & Hippel, 2010). Further, men who see other attractive men take greater financial risks to increase their desirability as a mating partner to women (Chan, 2015). Attractiveness-enhancement goals were found to increase young women’s willingness to take health-related risks such as tanning and taking dangerous diet pills (Hill & Durante, 2011). In addition, even though the few research that examined implicit theories of beauty (e.g. Burkley et al., 2014; Faust et al., 2024) did not directly test beauty-related risk-taking, the findings point to a possibility that an incremental (vs. entity) theory of beauty may be more related to risky beautification methods. Specifically, Faust et al. (2024) found that beauty entity (vs. incremental) theorists consume more cosmetic products, whereas Burkley et al. (2014) showed that beauty incremental (vs. entity) theorists were more likely to undertake cosmetic surgery, which is riskier compared to using cosmetic products. If a growth mindset of beauty leads to more beauty-related risk-taking, would it also influence risk-taking behavior beyond the beauty domain? Given the broad nature of beauty, we argue that should be the case.

We hypothesize that individuals who believe that beauty is malleable and improvable tend to take more risks in their decision-making because this belief may promote a sense of optimism about future outcomes in various domains in life. Why are beauty incremental theorists more optimistic than beauty entity theorists? Established research in implicit theories have shown that beliefs in malleability of human attributes shape individuals’ self-enhancement focus (Mathur et al., 2016). Fixed mindset disposes individuals to attend to signaling their performance and abilities to others whereas growth mindset disposes individuals to attend to learning of the relevant human attributes and traits (e.g. beauty, intelligence, morality). Entity theorists tend to give up more easily in order to avoid signaling poor performance to the self and others. In the domain of beauty, Faust et al. (2024) showed that entity theorists of beauty are similarly pressured about how they are evaluated by others. Beauty entity theorists experience social pressure arising from having to improve their physical appearance (i.e. to perform well). It suggests that this social pressure may hinder entity theorists from experiencing any optimistic feelings about future outcomes. In contrast, beauty incremental theorists tend to inherently focus on the improvement of beauty itself. Given the pervasive impact of beauty on social and professional success, we conjecture that believing in the improvability of beauty (i.e. incremental theory) may cultivate a sense of optimism about future outcomes in domains beyond beauty.

Indeed, as we mentioned earlier, beauty signals success in life. Beauty is positively correlated with perceptions of goodness (Tsukiura & Cabeza, 2011), trustworthiness (Wilson & Eckel, 2006), intelligence (Zebrowitz et al., 2002), and social skills (Langlois et al., 2000). Compared to unattractive adults, attractive adults receive more attention, positive social interaction, and help from others than do unattractive adults; they also achieve greater occupational success, have more dating experience, are more popular, and enjoy better physical and mental health (Langlois et al., 2000).

Previous research has shown that attractive faces elicit activation in brain regions that respond to other types of reward, such as monetary reward or pleasant chemosensory stimuli (Aharon et al., 2001). To the extent that people prefer beauty (e.g. choosing beautiful products; choosing to help beautiful beneficiaries) (Bloch et al., 2003; Cryder et al., 2017), merely believing that one’s own beauty can be improved may elevate a sense of positive outcome expectations. Beauty entity theorists, however, tend to focus on signaling their beauty (i.e. performance; or how good they look) to others; they may experience social pressure as recent studies suggest (Faust et al., 2024). Hence, believing that beauty is largely unimprovable does not dispose them to experience a similar sense of optimism.

A meta-analysis showed that a belief in incremental theory is positively associated with positive outcome expectations during self-monitoring of goal progress (Burnette et al., 2013). No research, however, has experimentally studied whether a more general sense of optimism can be generated and the causal influence of implicit theories on it. We propose that merely believing in the improvability of beauty may promote a sense of optimism that positive outcomes will occur in various domains of life. This might happen because of a belief held by many that beauty has broad impact in various areas. Our conjecture is supported by previous research, which suggested that physical attractiveness matters for important life outcomes (Anderson et al., 2008). Importantly, people believe that physically attractive individuals experience more positive life outcomes than do their unattractive counterparts (Dion et al., 1972). This effect has been documented in prior research as a physical attractiveness stereotyping effect, such that people not only judge attractive (vs. unattractive) individuals more positively on a variety of desirable attributes but also expect attractive individuals to experience greater overall happiness, obtain more prestigious jobs, have better marriages, and lead more satisfying social and professional lives (Dion et al., 1972). There seems to be an expectation that attractive people should achieve more desirable life outcomes, such as marital happiness and occupational success (Dickey-Bryant et al., 1986). Thus, believing one can improve one’s beauty should produce a sense of optimism that positive outcomes can be achieved in many domains of life.

This sense of optimism may motivate people to take more risks. Outside the beauty domain, a *correlational* relationship between optimism and risk-taking has already been established (Anderson & Galinsky, 2006; Puri & Robinson, 2007; Shah & Li, 2024). In a study by Anderson and Galinsky (2006), people who were primed with power had more optimistic risk perceptions which led them to take more risks. Previous research has shown that optimists (vs. pessimists) tend to focus on the positive (vs. negative) outcomes of risky decisions which leads them to take more risks (Dohmen et al., 2023). Even after experiencing negative gaming outcomes, optimists were more likely than pessimists to maintain positive expectations and continue gambling (Gibson & Sanbonmatsu, 2004). In an organizational context, there is evidence that CEOs who were optimists were more likely to take on and pay more for a corporate merger and were also more likely to undertake value-destroying mergers (Malmendier & Tate, 2008). Thus, the association between optimism and risk-taking is not new. No research, however, has examined the causal relationship between optimism and risk-taking in a seemingly unrelated domain. The present research intends to experimentally vary implicit theories of beauty and optimism to examine (a) whether implicit theories of beauty causally influence optimism that positive outcomes occur in *various* domains in life and, if so, (b) whether enhanced cross-domain risk-taking behavior results as a downstream consequence.

## Overview of Studies

Ten studies, including three supplementary studies, test our hypotheses. Across studies, we employed different measures of risk-taking behavior, both intentional and behavioral measures, in order to ensure that the effect observed is not merely a single effect on a specific risk-taking measure (Crandall & Sherman, 2016). More importantly, we intended to examine whether the effect of beauty implicit theories on risk-taking occurs in various seemingly unrelated domains of life, verifying our assumption that people hold a belief that beauty has broad impact on various areas of life.

In most studies, we aimed for at least 100 participants per condition. Results of sensitivity analyses by G*Power (Faul et al., 2009) show that these sample sizes provide 80% power (α = .05) to detect small effects in most studies (Study 1: *f*^2^ = .01; Study 2: *η*^2^ = .02; Study 3: *η*^2^ = .017; Study 4: *η*^2^ = .024; Study 5: *d* = 0.19; Study 6: *d* = 0.39; Study 7: *η*^2^ = .027). We report all manipulations, measures, and exclusions in these studies. Studies 5, 6, 7, and Supplementary Studies B and C were preregistered.^1^ Data, analysis codes, and materials are available at https://osf.io/4s3nw/?view_only=07b74c9e89104d3e9458982b7725b18d.

## Study 1

Study 1 examined the proposed effects across cultures in the context of financial risk-taking. This focus is important because it enables a verification of the pervasiveness of beauty and beauty mindsets across cultures. We considered samples from the United States and India as these two countries vary on the individualism–collectivism dimension (Hofstede, 1980; Triandis & Gelfland, 1998). Previous research has shown that individuals in a collectivist society are less risk-averse than those in an individualistic society because they are more likely to receive financial help if they need (i.e. the cushion hypothesis; Hsee & Weber, 1999). Moreover, there exists similarities in attitude toward perceived risk in these cultures (Weber & Hsee, 1998). It is therefore important to test if our hypothesized effect of beauty implicit theories may be related to these cultural influences.

### Methods

A total of 1,057 participants on Amazon Mechanical Turk from the United States (*n*_US_ = 552; 44.7% females, *M*_age_ = 39.61, *SD* = 14.23) and from India (*n*_India_ = 505; 34.9% females, *M*_age_ = 31.72, *SD* = 7.04) were recruited. Similar recruitment approach has been used in previous cross-cultural research (Gruda & Kafetsios, 2020; Tripathi et al., 2018).

We adopted Faust et al.’s (2024) measure of implicit theories of beauty, with three items: “You have a certain amount of beauty and you can’t do much to change it,” “Your beauty is something about you that you can’t change very much,” and “You can enhance your appearance, but you can’t really change your basic beauty” (1 = *strongly agree*, 7 = *strongly disagree*; endpoints adapted from Chiu, Hong, et al., 1997). An average of the three items serves as implicit theories index, with the higher score indicating greater incremental belief (Chiu, Hong, et al., 1997). Faust et al. (2024) developed these items based on the established, validated measure of implicit theories of intelligence, morality, and personality (Dweck et al., 1995).

Following this measure, we included 14 filler items (e.g. I like watching TV). We then measured risk-taking by having participants indicate their likelihood of engaging in eight behaviors (e.g. “Betting a day’s income at the horse races”) (1 = *very unlikely*, 7 = *very likely*; all items in Supplemental Material S1; Weber et al., 2002).

Further, we measured culture orientation, with 16 items to measure four dimensions of vertical/horizontal collectivism/individualism (Triandis & Gelfland, 1998; Supplemental Material S2). Finally, we captured demographic variables including participants’ age and gender.

### Results

To check for common method bias, we used the common latent factor method which involves using a common latent factor (CFL) to capture the common variance among all observed variables in a confirmatory factor analysis mode (Gaskin, 2012). A comparison of standardized regression weights between the models with (vs. without) the CLF showed large differences (>.2) for horizontal and vertical collectivism items. Thus, we retained the CLF as we imputed composites from factor scores. We then used the common variance method-adjusted composites to run regression analyses.

**Figure 1.**
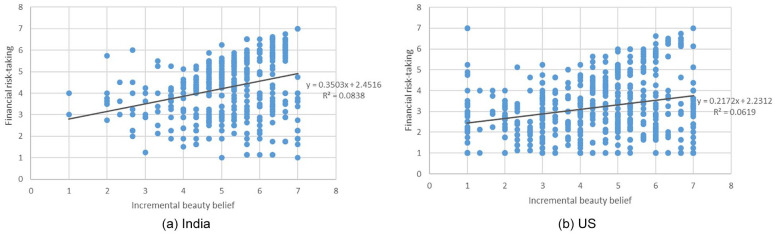
Study 1—Correlation between implicit theories of beauty and risk-taking in India and the United States.

We conducted a linear regression analysis with implicit theories of beauty, country, and country*theory interaction term as factors, risk-taking as the dependent variable, and the four dimensions of culture orientation as covariates (see Correlation matrices in Supplemental Material S3). The overall model was significant (*F*(7, 1049) = 61.91, *p* < .001, *f*^ 2^ = .41). Results revealed a significant positive effect of beauty implicit theories on risk-taking (*β* = .47, 95% CI [0.61, 0.90], *t*(1,049) = 9.90, *p* < .001), and a significant main effect of country (*β* = −.22, 95% CI [−1.07, −0.62], *t*(1,049) = −7.39, *p* < .001). The interaction of country and beauty implicit theories was significant (*β* = −0.15, 95% CI [−0.47, −0.11], *t*(1,049) = −3.16, *p* = .002). In both India (*β* = .38, 95% CI [0.59, 0.90], *t* = 9.61, *p* < .001) and the United States (*β* = .35, 95% CI [0.35, 0.55], *t* = 8.71, *p* < .001), the effect of beauty implicit theories on risk-taking was significant. Thus, while the effect seems to be stronger in India than the United States, the effect is present in both countries (see Figure 1). Please refer to Table 1 and Table S1 in Supplemental Material S3 for the results of the culture orientation dimensions. Further, we conducted additional analyses to understand whether participants’ gender has any influence on the theory-risk-taking effect. Results showed a significant interaction effect of gender and beauty implicit theories on risk-taking. We reported the detailed results in Supplemental Material S19.

**Table 1.** Regression Results of Culture Orientation Dimensions.

Culture orientation dimension	Regression results
Horizontal collectivism	*β* = .16, 95% CI [1.65, 3.58], *t*(1,049) = 5.32, *p* < .001
Vertical collectivism	*β* = −.11, 95% CI [−4,388.34, −1,270.87], *t*(1,049) = −3.56 *p* < .001
Horizontal individualism	*β* = .05, 95% CI [−0.01, 0.48], *t*(1,049) = 1.88, *p* = .06
Vertical individualism	*β* = .11, 95% CI [0.08, 0.30], *t*(1,049) = 3.47, *p* < .001

### Discussion

Results from the cross-country survey provide initial evidence that an incremental theory of beauty is associated with greater risk-taking tendency. The correlation was consistent in both countries, the United States and India, and remained significant when controlling for cultural orientation dimensions (i.e. horizontal/vertical horizontalism/individualism). This result suggests that the relationship of beauty implicit theories and risk-taking exists and appears to be pervasive across these two cultures. The effect of beauty implicit theories on financial risk-taking appears to be stronger in India than in the United States. We did not hypothesize the latter a priori. This, however, may provide valuable insights for future research. Next, to test for a causal effect of implicit theories of beauty on risk-taking, we experimentally manipulated implicit theories in the next studies.

## Study 2

### Methods

#### Participants and Design

Three hundred and fifty-nine respondents from Amazon Mechanical Turk (53.8% females, *M*_age_ = 31.88, *SD* = 10.76) were randomly assigned to one of the two conditions in a one-factor (Implicit theories of beauty: entity vs. incremental) between-subjects design.

#### Procedure

To manipulate implicit theories, we adapted a well-established manipulation in the intelligence domain (Bergen, 1991; Hong et al., 1999). In a task labeled “Reading Comprehension Task,” we showed participants a fictitious article in *Time* magazine. The entity (incremental) belief article highlighted the idea that beauty cannot be improved (beauty is malleable; Supplemental Material S4a). To reinforce the manipulation participants were asked to summarize the main theme of the article. We pretested this implicit theory manipulation (Supplemental Material S5a).

To assess real risk-taking behavior, we used a well-established measure, the Balloon Analogue Risk Task (BART) (Lejuez et al., 2002). Participants played a computer-based game in which they pumped air into balloons. For each pump, they received one point; however, if the balloon popped before being released, all the points for that particular balloon would be lost. The game determined randomly if a balloon ruptured or not. One way to keep the points is to release the current balloon and start a new one; as such, more risk-averse individuals tend to bank their points after fewer pumps. Each participant pumped air into a total of 30 balloons (i.e. trials).

To make this measure consequential, we informed participants that if they received the highest points among all participants, they would get a $50 Amazon gift card. As suggested by Lejuez et al. (2002), a higher number of pumps on unexploded balloons is indicative of greater risk-taking propensity.

Several control variables were considered, including (a) participants’ perception of their own beauty since it might affect how implicit theories influence them (Hong et al., 1999), (b) participants’ perceptions of the effectiveness of different beautification methods, (c) participants’ perceived importance of being beautiful, (d) chronic, trait risk-taking, and (e) mood (please see Supplemental Material S6 for the rationale of including these control variables, and Supplemental Material S7 for all measures).

As a manipulation check, participants indicated their belief in the fixedness of beauty using the same three items as in Study 1 (α = .91). Lastly, participants indicated their demographic information (i.e. age and gender) and were debriefed.

### Results

#### Manipulation Check

An independent t-test confirmed that perceived fixedness of beauty was higher in the entity (vs. incremental) theory condition (*M*_entity_ = 4.41, *SD* = 1.66 vs. *M*_incremental_ = 3.63, *SD* = 1.64; *t*(348) = −4.43, *p* < .001, *d* = 0.47). In the subsequent studies, our implicit theories manipulation check showed similar results. We report them in Supplemental Material S9.

#### Risk-taking Behavior

An ANOVA treating the number of pumps on unexploded balloons as the dependent variable and implicit theories of beauty (entity vs. incremental) as the independent variable revealed a significant main effect of beauty beliefs on risk-taking. An incremental (vs. entity) belief led to a higher number of pumps on unexploded balloons (*M*_entity_ = 446.99, *SD* = 244.60 vs. *M*_incremental_ = 533.29, *SD* = 242.37; *F*[1, 357] = 11.27, *p* < .001, *η*^2^ = .03).

#### Control Variables

Independent t-tests showed no significant differences between beauty entity and incremental theorists in all control variables (see Supplemental Material S8 for the results). Further, an ANCOVA with the number of pumps on unexploded balloons as the dependent variable, implicit theories as the independent variable, and these control variables as covariates showed that the effect of beauty implicit theories remained significant (*F*[1, 335] = 11.44, *p* < .001, *η*^2^ = .03). These patterns of results were similar in the next studies (Studies 3 and 4; reported in Supplemental Materials S12 and S13, respectively). As such, we will not discuss them further.

Moreover, additional analyses (reported in Supplemental Material S19) revealed no significant interaction effect of gender and beauty implicit theories on risk-taking.

### Discussion

Overall, results from experiment 2 showed that regardless of the extent to which participants perceived themselves to be beautiful, holding an incremental (vs. fixed) theory of beauty promotes risk-taking. Further, the effect held controlling for individuals’ trait risk-attitude, suggesting that implicit theories of beauty can influence risk-taking regardless of one’s chronic attitudes toward taking risks. We demonstrated the effect of beauty implicit theories on risk-taking with a real behavioral measure (i.e. monetary payoffs) of risk-taking. Further, to provide more support for our hypothesis, we conducted Supplementary Study A (reported in Supplemental Material S10) in which we replicated the effect with a different measure of risk-taking. It is however unclear whether this effect was driven by an enhancing effect of incremental theory or an attenuating effect of entity theory. This question is worth our attention because previous research has shown that beauty entity theorists tend to experience social pressure to improve beauty (Faust et al., 2024). Thus, in experiment 3, we included a control condition.

## Study 3

### Methods

#### Participants and Design

A total of 551 participants (56.9% females, *M*_age_ = 33.51, *SD* = 10.20) from Cloud Research were randomly assigned to one of the three conditions in a one-factor, three-level (Implicit theories: entity vs. incremental vs. control) between-subjects design.

#### Procedure

The measures and procedures were similar to those in experiment 2, with two exceptions. First, we included a control condition in which participants read an article unrelated to beauty (i.e. the properties of water) (Tadmor et al., 2013). Second, we used a different risk-taking measure. Following Hsee and Weber (1999) and Levav and Argo (2010), our task required making a series of 14 hypothetical choices between a cash payoff and a risky gamble that offered a 50% chance of winning a bigger cash prize or nothing (e.g. “receive $400 for sure” vs. “flip a coin; receive $2,000 if Heads or $0 if Tails”; Supplemental Material S11). The total number of risky choices across the 14 choices served as our dependent variable.

### Results

**Figure 2.**
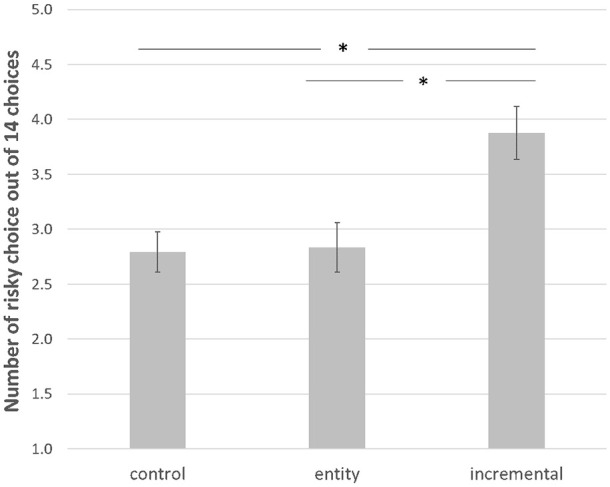
Experiment 3—Number of risky choices as a function of implicit theories of beauty. *Note*. Error bars represent Standard Errors. *p < .05.

Results from an ANOVA with implicit theories of beauty (entity vs. incremental vs. control) as a single factor on the number of riskier choices across the 14 options revealed a significant effect (*F*[2, 548] = 7.84, *p* < .001, *η*^2^ = .03). A post-hoc Tukey’s HSD test showed that participants in the incremental belief condition chose the risky choice more frequently than participants in the entity belief (*M*_incremental_ = 3.88, *SD* = 3.19 vs. *M*_entity_ = 2.83, *SD* = 3.07, *p* = .002, *d* = 0.34) and control condition (*M*_incremental_ = 3.88, *SD* = 3.19 vs. *M*_control_ = 2.79, *SD* = 2.53, *p* = .001, *d* = 0.38) (Figure 2). The difference in the number of riskier choices between the entity and control conditions was not significant (*M*_entity_ = 2.83, *SD* = 3.07 vs. *M*_control_ = 2.79, *SD* = 2.53, *p* = .992, *d* = 0.01). Further, additional analyses showed that there was a significant interaction effect of gender and beauty implicit theories (incremental vs. entity vs. control) on the number of riskier choices (see Supplemental Material S19).

### Discussion

Experiment 3 demonstrated that the effect observed is driven by the incremental theory of beauty which increased risk-taking, rather than by entity theory reducing risk-taking. Specifically, participants in the control condition were equally likely to choose the riskier choices as entity theorists and were less likely to do so compared with incremental theorists. These findings also rule out an alternative explanation that the effects we observed are driven by perceived pressure experienced by participants. Recent studies show that entity theorists experience more pressure about changing their physical appearance than incremental theorists (Faust et al., 2024). If perceived pressure is the underlying mechanism, we should have observed that an entity belief decreases risk-taking compared to the control and incremental belief conditions. We do not observe any negative impact of beauty entity theory on risk-taking. We speculate that to the extent that entity theorists experience very intense social pressure, this negative feeling may dampen their risk-taking propensity. This (latter effect of why there is no negative effect compared to the control condition) is outside the focus of the present research, and we will not discuss it further.

So far our findings suggest that believing in the improvability of beauty led people to take more risks. However, one might argue that the malleable nature suggested in incremental theory (vs. entity theory) may itself promote risk-taking independent of whether it is related to the beauty domain or not. To examine this possibility, in experiment 4, we orthogonally manipulated implicit theories and domains—beauty versus intelligence. We tested two competing hypotheses: (a) incremental theories promote risk-taking, regardless of whether it is about intelligence or beauty, and (b) the incremental theory of beauty promotes risk-taking. If the former hypothesis is true, we should observe a main effect of incremental (vs. entity) theory. If the latter is true, we should observe an interaction of implicit theories and domains, in which the effect of incremental (vs. entity) theory on risk-taking is valid only when the domain is beauty.

## Study 4

### Methods

#### Participants and Design

Four hundred and thirty-nine participants on Amazon Mechanical Turk (53.3% females, *M*_age_ = 33.24, *SD* = 10.17) were randomly assigned to one of the four conditions in a 2 (Implicit theories: entity vs. incremental) × 2 (Domain: beauty vs. intelligence) between-subjects design.

#### Procedure

We manipulated implicit theories as in experiments 2 and 3. The intelligence articles were quite identical to the beauty articles, with the exception of the domain.

To measure risk-taking, participants made a decision between two lottery options in two scenarios :“an 80% chance of winning $200 and a 20% chance of winning nothing” versus “a 20% chance of winning $800 and an 80% chance of winning nothing” (Duclos et al., 2013), and “50% chance of winning $50 and 50% chance of winning $100” versus “50% chance of winning $20 and 50% chance of winning $130” (Esteky et al., 2018) (1 = *strongly prefer option A*, 7 = *strongly prefer option B*). In both scenarios, option B was riskier. Thus, a higher score indicates higher risk-taking. We averaged the scores in the scenarios as a risk-taking index.

Manipulation checks were then administered. Similar to the implicit theories of beauty measure, our manipulation check for implicit theories of intelligence included three items (see Supplemental Material S9).

### Results

**Figure 3.**
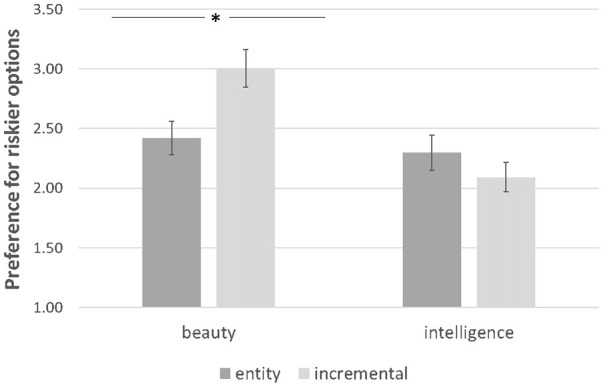
Experiment 4—Preference for risky options as a function of implicit theories of beauty and intelligence. *Note.* Error bars represent Standard Errors. *p < .05.

A 2 (Implicit theories: entity vs. incremental) × 2 (Domain: beauty vs. intelligence) ANOVA with risk-taking as the dependent variable yielded a non-significant main effect of implicit theories (*F*[1, 423] = 1.79, *p* = .18, *η*^2^ = .004), and a significant main effect of domain (*M*_beauty_ = 2.71, *SD* = 1.55 vs. *M*_intelligence_ = 2.19, *SD* = 1.42; *F*[1, 423] = 13.08, *p* < .001, *η*^2^ = .03). More importantly, a significant interaction of implicit theories and domain was revealed (*F*[1, 423] = 7.69, *p* = .006, *η*^2^ = .02; Figure 3). In beauty conditions, incremental (vs. entity) theorists showed a higher preference for the risky options (*M*_incremental beauty_ = 3.00, *SD* = 1.62 vs. *M*_entity beauty_ = 2.42, *SD* = 1.43; *F*[1, 423] = 8.32, *p* = .004, *η*^2^ = .02). In intelligence conditions, the effect disappeared (*M*_incremental intelligence_ = 2.09, *SD* = 1.34 vs. *M*_entity intelligence_ = 2.30, *SD* = 1.50; *F*[1, 423] = 1.05, *p* = .31, *η*^2^ = .002). Further, additional analyses revealed a non-significant interaction effect of gender and beauty implicit theories on risk-taking (Supplemental Material S19).

### Discussion

Results from Study 4 demonstrated that incremental (vs. entity) theorists of beauty exhibited greater risk-taking. Implicit theories of intelligence, on the other hand, did not have any effect on risk-taking. These results suggest that the effects of implicit theories of beauty on risk-taking behavior observed across four studies thus far were not driven by the nature of malleability per se. In the next studies, we move on to examine the underlying mechanism. Study 5 provided initial evidence that beauty is implicitly associated with optimism. Study 6 tested the mediating role of the optimism that positive outcomes will occur in various domains of life. Study 7 demonstrated the role of the belief that beauty has broad impact by directly manipulating it. In addition, we report two supplementary studies B and C in Supplemental Materials S14 and S15, in which we used a causal-chain mediation design to test the role of a general sense of optimism in the effect of beauty implicit theories on risk-taking behavior. Specifically, Supplementary Study B established the causal link between implicit theories of beauty and optimism, whereas Supplementary Study C directly manipulated optimism to test for its causal effect on risk-taking behavior.

## Study 5

As the first step to demonstrate that believing one can improve their beauty leads to optimism, in this study (preregistration: https://aspredicted.org/2cry-69q7.pdf) we aimed to provide evidence that beauty is implicitly associated with optimism. To this end, we employed an Implicit Association Test to show that being beautiful is more strongly associated with optimism than being moderate-looking.

### Methods

Two hundred and one U.S. participants from Prolific (43.8% females, *M*_age_ = 40.1, *SD* = 13.57) participated in the study. We designed a Single Category Implicit Association Test (SC-IAT) following the approach by Karpinski and Steinman (2006) to measure the association of beauty (vs. moderate-looking) and optimism. The SC-IAT consisted of two stages, which all participants completed in the same order. Each stage consisted of 24 practice trials immediately followed by 72 test trials (three blocks of 24 trials each) (see Table 2 for a summary). In the first stage (beauty + optimism), beauty and optimism words were categorized on the E key, and moderate-looking words were categorized on the I key. In the second stage (moderate-looking + optimism), beauty words were categorized on the E key, and moderate-looking and optimism words were categorized on the I key. Six words were used for each category—beauty (beautiful, gorgeous, good-looking, attractive, pretty, stunning), moderate-looking (average-looking, moderately attractive, ordinary looking, intermediately attractive, mediocre-looking, plain-looking), and optimism (optimistic, hopeful, positive, bright, rose-colored, auspicious). Within each category, words were selected randomly. Each target word appeared centered on the screen and remained on the screen until the participant responded or for 1,500 ms. If participants failed to respond within 1,500 ms, a reminder to “Please respond more quickly!” appeared for 500 ms. Following each response, a green O (a red X) appeared in the center of the screen if the response was correct (incorrect).

**Table 2.** Summary of the IAT Task in Study 5.

Block	Trials	Function	Left-key response	Right-key response
1	24	Practice	Beauty words + optimism words	Moderate-looking words
2	72	Test	Beauty words + optimism words	Moderate-looking words
3	24	Practice	Beauty words	Moderate-looking words + optimism words
4	72	Test	Beauty words	Moderate-looking words + optimism words

The D-score provided by the IAT program served as our dependent variable. This D-core algorithm is calculated by Inquisit, which does not included data from the practice trials (blocks 1 and 3) and excluded responses less than 350 ms (Karpinski & Steinman, 2006). In this SC-IAT, a positive D-score would indicate a stronger beauty-optimism association, whereas a negative D-score would indicate a stronger moderately looking—optimism association.

### Results and Discussion

A one-sample t-test comparing the average D-score with 0 showed that the D-score is significantly larger than 0 (*M* = 0.05, *SD* = 0.34, *t*(200) = 2.07, *p* = .04, *d* = 0.15). This result confirms that people have an automatic association between beauty and optimism. Further, an independent t-test showed that there is no difference in D-score between men and women (*p* = .70), indicating that both men and women similarly hold a beauty-optimism association.

## Study 6

### Methods

#### Participants and Design

In this study (preregistration: https://aspredicted.org/63dc-by6c.pdf), 203 participants on Prolific (60.6% females; *M*_age_ = 43.72, *SD* = 14.50) were randomly assigned to one of the two conditions in a single-factor 2 (Implicit theories of beauty: entity vs. incremental) between-subjects design.

#### Procedure

Participants completed the implicit theories of beauty manipulation in the same way as in previous studies, although the article was a shorter version (see Supplemental Material S4b, pretest in Supplemental Material S5b). We then measured risk-taking with four items (adopted from White et al., 2024). Participants were asked: “If you have the opportunity, how likely are you to engage in the following behaviors within the next month?” for example, “Hiking a potentially unsafe trail or going down a potentially dangerous ski slope” (1 = *extremely unlikely*, 7 = *extremely likely*; the same items as in Supplementary Study A, see Supplemental Material S10; α = .65). Then, we measured optimism in many domains of life with four items, adapted from Cheung et al. (2013) (e.g. “I am feeling optimistic about my future in many domains of life”; 1 = *strongly disagree*, 7 = *strongly agree*; all items in Supplemental Material S16; α = .93). Finally, we included the manipulation check for implicit theories of beauty (Supplemental Material S9), and demographic variables as before.

### Results

One participant did not provide a meaningful summary of the article in our implicit theory manipulation. As preregistered, we excluded this participant from final analyses.

#### Risk-taking

An independent *t*-test showed that beauty incremental theorists (*M* = 2.87, *SD* = 1.32) indicated greater risk-taking compared to entity theorists (*M* = 2.36, *SD* = 1.02; *t*(200) = 3.03, *p* = .003, *d* = 0.43).

#### Optimism in Many Domains of Life

An independent t-test also showed that beauty incremental theorists (*M* = 4.97, *SD* = 1.43) showed greater optimism in many domains of life compared to entity theorists (*M* = 4.22, *SD* = 1.56, *t*(200) = 3.60, *p* < .001, *d* = 0.51).

#### Mediation Analysis

We conducted a mediation analysis using Hayes’ Process model 4 (Hayes, 2017) with beauty theories as the independent variable, risk-taking as the dependent variable, and optimism in many domains of life as the mediator. Results showed that implicit theories of beauty had a significant effect on optimism (*B* = 0.76, *SE* = 0.21, *t* = 3.60, *p* = .0004), optimism in turn significantly influenced risk-taking (*B* = 0.19, *SE* = .05, *t* = 3.43, *p* = .0007). The indirect effect of beauty theories on risk-taking through optimism was significant (*B* = 0.14, *SE* = 0.06, 95% CI [0.04, 0.26]). The direct effect of beauty theories on risk-taking was still significant (*B* = 0.36, *SE* = 0.17, *t* = 2.17, *p* = .03), suggesting that the mediation of optimism in many domains of life was partial.

Further, additional analyses showed that beauty theories and gender did not interactively influence optimism and risk-taking (see detailed results in Supplemental Material S19).

### Discussion

Results from this study showed that compared to an entity theory of beauty, an incremental theory of beauty was associated with greater optimism about positive outcomes in many domains of life. This optimism was associated with greater risk-taking. A limitation of this study is that the mediator (i.e. optimism) was measured and not experimentally manipulated. In such a measurement-of-mediation design, causal evidence is uncertain (Laghaie & Otter, 2023). To address this issue, in Study 7, we manipulated the mediator. As we argued before, the optimism experienced by beauty incremental theorists stems from a belief that beauty has broad impact in various areas of life. Thus, in Study 7, we directly manipulated this belief.

## Study 7

In Study 7 (preregistration: https://aspredicted.org/ygbx-478t.pdf), using a process-by-moderation approach, we aim to test our prediction that the effect of beauty implicit theories on risk-taking is moderated by the belief that beauty has broad impact in various domains of life. If this prediction is correct, the effect should disappear when the belief that beauty is broad is weakened.

### Methods

#### Participants and Design

Four hundred and one participants from the United States on Prolific (54.4% females, *M*_age_ = 40.81, *SD* = 13.29) were randomly assigned to one of the four conditions in a 2 (Implicit theories of beauty: entity vs. incremental) × 2 (“Beauty is broad” belief: strong vs. weak).

#### Procedure

We used the same manipulation for beauty implicit theories as in previous studies. To manipulate “beauty is broad” belief, we employed a writing task. Specifically, participants read that “Some people believe that beauty is linked to many domains of life. That is, beauty has broad impact on various areas.” Participants in the strong (vs. weak) belief condition were asked to provide 8 (vs. 2) examples to support this view. This manipulation was confirmed to be effective by a pretest (see Supplemental Material S18).

Following this belief manipulation, we measured risk-taking using the same items as in Study 6. Finally, we included a manipulation check for implicit theories of beauty as in previous studies.

### Results

As preregistered, we excluded eight participants who did not provide a meaningful summary of the beauty article. We note that without exclusions the pattern of results remained the same.

**Figure 4.**
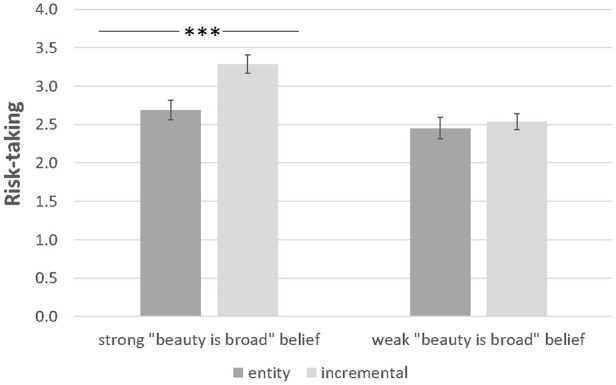
Experiment 7—Risk-taking as a function of implicit theories of beauty and “beauty is broad” belief. *Note.* Error bars represent Standard Errors. ***p < .001.

We conducted a 2 (Implicit theories of beauty: entity vs. incremental) × 2 (Beauty is broad belief: strong vs. weak) ANOVA on risk-taking. Results revealed a significant main effect of beauty implicit theories (*M*_entity_ = 2.57, *SD* = 1.24 vs. *M*_incremental_ = 2.91, *SD* = 1.25; *F*[1, 389] = 7.71, *p* = .006, *η*^2^ = .02), a significant main effect of beauty is broad belief (*M*_strong belief_ = 2.99, *SD* = 1.34 vs. *M*_weak belief_ = 2.50, *SD* = 1.11; *F*[1, 389] = 15.93, *p* < .001, *η*^2^ = .04), and importantly, a significant interaction effect of beauty implicit theories and beauty is broad belief (*F*[1, 389] = 4.33, *p* = .038, *η*^2^ = .01) (Figure 4). Simple-effects tests showed that when participants strongly believed that beauty is broad (i.e. when they wrote eight examples), beauty incremental (vs. entity) theorists indicated greater risk-taking (*M*_strong belief/incremental_ = 3.29, *SD* = 1.35 vs. *M*_strong belief/entity_ = 2.69, *SD* = 1.27; *F*[1, 389] = 11.77, *p* < .001, *η*^2^ = .03). When the belief that beauty is broad is weakened (i.e. participants wrote two examples), this effect disappeared (*M*_weak belief/incremental_ = 2.54, *SD* = 1.03 vs. *M*_weak belief/entity_ = 2.45, *SD* = 1.20; *F*[1, 389] = .24, *p* = .62, *η*^2^ = .001). Further, additional analyses with gender as a factor showed that the three-way interaction of theories, beauty is broad belief, and gender was not significant (refer to Supplemental Material S19 for the details).

### Discussion

Results from this study confirmed that the effect of implicit theories of beauty on risk-taking is driven by the belief that beauty has broad impact in many domains of life. When this belief was weakened, the effect disappeared. Together with findings from Study 6, this provided evidence that believing beauty can be improved (i.e. an incremental theory of beauty) produces the optimism that one will achieve positive outcomes in many domains of life, because beauty has broad impact in various areas. This latter finding is consistent with previous research which showed that beauty is positively correlated with many other attributes such as trustworthiness (Wilson & Eckel, 2006), intelligence (Zebrowitz et al., 2002), perceptions of goodness (Tsukiura & Cabeza, 2011), and social skills (Langlois et al., 2000).

## General Discussion

Ten studies, including three supplementary studies, support the core hypothesis that implicit theories of beauty influence people’s tendency to engage in risk-taking behavior. Study 1 provided cross-country evidence on the correlational relationship between implicit theories of beauty and risk-taking. Study 2 provided causal evidence for the effect of beauty theories on a real, consequential risk-taking behavior. Supplementary Study A replicated this effect with a different measure of risk-taking. In Study 3, including a control condition indicated that it is the enhancing effect of an incremental theory, rather than the attenuating effect of an entity theory, that drove the risk-taking tendency observed. In Study 4, we showed that the effect of implicit theories is contingent on the domain; implicit theories of beauty influenced people’s risk-taking tendency, whereas implicit theories of intelligence did not show any effects. In Study 5, we showed that people hold a strong implicit association between beauty and optimism, more so than between moderate-looking and optimism. In Studies 6 and 7, we demonstrated that the effect is specific to beauty because of the belief that beauty has broad impact in various areas of life. Specifically, Study 6 showed the mediation of felt optimism that positive outcomes will occur in many domains of life, and Study 7 showed that the effect disappeared when the belief that beauty has broad impact was weakened. Supplementary Studies B and C further confirmed that optimism underlined the effect of implicit theories of beauty on risk-taking. Supplementary Study B established the causal link between implicit theories of beauty and optimism, and Supplementary Study C directly manipulated optimism to demonstrate its causal effect on risk-taking behavior.

Previous research has documented how implicit theories may influence information processing and decision-making (Dweck et al., 1993, 1995; Dweck & Leggett, 1988; Kwon & Nayakankuppam, 2015; Mathur et al., 2012). Most of the existing findings have largely focused on understanding the impact of implicit theories on domain-specific decision contexts. To our knowledge, the present research is among the first to demonstrate that domain-specific implicit theories may influence decision-making and behavior outside that domain. As noted earlier, we consider cross-domains of life that are seemingly unrelated to or not directly related to beauty. We seek to extend past research on implicit theories by demonstrating the effect of implicit theories of beauty on an important aspect of human behavior, namely risky decision-making. Adding to previous research which demonstrated that risk-taking in decision-making can be affected by cognitive, emotional, and social factors that are not directly related to the decision per se (e.g. Duclos et al., 2013; Hsee & Weber, 1999; Zhu et al., 2012), we showed that implicit theories of beauty is a novel and important antecedent of risk-taking behavior. Further, this work is the first to demonstrate that (a) implicit theories of beauty increases optimism that positive outcomes would occur in various domains in life, and (b) this sense of optimism increases risk-taking behavior. We thus contribute to the literature examining the antecedents (Anderson & Galinsky, 2006; Monga & Houston, 2006) and consequences of optimism (Chan et al., 2013; Zhang et al., 2007).

Our findings ruled out several alternative explanations including salience of loss in financial decision scenarios (Montford et al., 2019), perceptions of one’s own beauty, cultural orientations, and affect. In particular, Study 1 showed that the correlational relationship between implicit theories of beauty and risk-taking remains across cultures that is more collectivistic or individualistic. Across studies, we considered both decision-making scenarios in which loss is salient or not so salient. A previous research showed that loss salience moderates the impact of implicit self-theories on financial risk-taking (Montford et al., 2019). The latter work focuses on a different domain and a different dependent variable (self-theories, rather than beauty theories; and financial risk-taking, rather than general risk-taking as in the present research). Our results suggest that beauty implicit theories influence risk-taking behavior regardless of salience of loss presented in the decision scenarios considered.

Our findings also provide important societal implications. By identifying the consequence of implicit beauty beliefs and shedding light on the mechanism and motivation behind it, we hope to help individuals make more informed decisions in their lives. For instance, individuals who tend to believe in the malleability of beauty should be aware of how their heightened optimism may affect their decisions and thus, such individuals should especially consider their choices carefully and rationally to avoid any unrealistic optimism.

The present research informs several meaningful avenues of future research. First, while we found that an incremental theory of beauty increased the optimism that one will achieve favorable outcomes in many domains of life, we did not directly test why this occurs. Although the impact of implicit theories on information processing and decision-making is well-established (Dweck et al., 1993, 1995; Dweck & Leggett, 1988), its impact on optimism is not foreordained. It is plausible that individuals who endorse a beauty incremental belief would perceive a heightened likelihood of attaining mastery of beauty, which in turn may enhance a sense of optimism of future positive outcomes given the broad nature of beauty. Future research may examine this assumption. Second, we did not investigate any potential boundary conditions. Our conjecture is that beauty implicit theories have an impact on risk-taking in domains outside of beauty (cross-domains of life). We showed empirical evidence for our predictions across various domains. However, we did not examine the boundaries of our conjecture. Future research can further examine potential moderators that could influence the effect of beauty theories on risk-taking behavior of different domains. Third, numerous research has shown that gender plays a significant role in the impact of physical attractiveness (Feingold, 1990; Li et al., 2013; Shackelford et al., 2005). To examine the role of gender in the effect of beauty implicit theories on risk-taking in our studies, we conducted additional analyses (reported in Supplemental Material S19) which showed that the effects of gender were not consistent across studies. For example, in Study 1 in which we measured chronic beauty implicit theories and risk-taking propensity across two countries, we observed a significant interaction of beauty implicit theories and gender on risk-taking. The effect of beauty incremental theory was stronger among men than women. In Study 3, we observed similar interactive effect of beauty implicit theories and gender. In other studies, beauty implicit theories did not interact with gender. We do not know why this is the case. Given the gender differences demonstrated in the attractiveness literature, research investigating the role of gender in the effects of beauty implicit theories could offer intriguing insights.

## Supplemental Material

sj-docx-1-psp-10.1177_01461672251327605 – Supplemental material for The Growth Mindset of Beauty Promotes Risk-Taking Propensity and Behavior Supplemental material, sj-docx-1-psp-10.1177_01461672251327605 for The Growth Mindset of Beauty Promotes Risk-Taking Propensity and Behavior by Natalie T. Faust and Iris W. Hung in Personality and Social Psychology Bulletin

## Notes

1. See Supplemental Materials S17 for deviation from preregistration in Study 7.
