# Supplementary material for: The Growth Mindset of Beauty Promotes Risk-Taking Propensity and Behavior
Source: Pers Soc Psychol Bull. 2025 Apr 2;52(7):1903–16. doi: 10.1177/01461672251327605 (PMC13216566; doi:10.1177/01461672251327605)
Supplement: sj-docx-1-psp-10.1177_01461672251327605 – Supplemental material for The Growth Mindset of Beauty Promotes Risk-Taking Propensity and Behavior [file sj-docx-1-psp-10.1177_01461672251327605.docx]

**Supplementary Materials**

**The Growth Mindset of Beauty Promotes Risk-Taking Propensity and Behavior**

All data, code, and materials for this manuscript can be found here: <https://osf.io/zr79g/?view_only=61b595aefa4142f8b70e255828b3c904>

**Supplementary Material S1. Trait risk-taking attitude measure (**Weber et al., 2002)

Betting a day’s income at the horse races

Investing 10% of your annual income in a moderate growth mutual fund

Betting a day’s income at a high stake poker game

Investing 5% of your annual income in a very speculative stock

Betting a day’s income on the outcome of a sporting event (e.g., baseball, soccer, or football)

Investing 5% of your annual income in a conservative stock

Investing 10% of your annual income in government bonds (treasury bills)

Gambling a week’s income at a casino

*(1 = not likely at all; 7 = very likely)*

**Supplementary Material S2. Cultural Orientation measure (**Triandis & Gelfland, 1998)

Horizontal individualism items:

1. I'd rather depend on myself than others.

2. I rely on myself most of the time; I rarely rely on others.

3. I often do "my own thing."

4. My personal identity, independent of others, is very important to me.

Vertical individualism items:

1. It is important that I do my job better than others.

2. Winning is everything.

3. Competition is the law of nature.

4. When another person does better than I do, I get tense and aroused.

Horizontal collectivism items:

1. If a coworker gets a prize, I would feel proud.

2. The well-being of my coworkers is important to me.

3. To me, pleasure is spending time with others.

4. I feel good when I cooperate with others.

Vertical collectivism items:

1. Parents and children must stay together as much as possible.

*2.* It is my duty to take care of my family, even when 1 have to sacrifice what I want.

3. Family members should stick together, no matter what sacrifices are required.

4. It is important to me that I respect the decisions made by my groups.

*(1 = Never or definitely no, 7 = Always or definitely yes)*

**Supplementary Material S3. Correlation matrix in Study 1**

*Table S1. Correlation matrix for both countries (US and India) collapsed*

|  | | **1** | **2** | **3** | **4** | **5** | **6** |  |  |
| --- | --- | --- | --- | --- | --- | --- | --- | --- | --- |
| **1. Horizontal collectivism** | | - |  |  |  |  |  |  |  |
| **2. Vertical collectivism** | | .329^***^ | - |  |  |  |  |  |  |
| **3. Vertical individualism** | | -.358^***^ | -.254^***^ | - |  |  |  |  |  |
| **4. Horizontal individualism** | | -.035 | .266^***^ | .130^***^ | - |  |  |  |  |
| **5. Risk taking** | | .067^*^ | -.100^***^ | .236^***^ | .030 | - |  |  |  |
| **6. Theory** | | -.031 | .023 | .158*** | .024 | .425*** | - |  |  |
| ****p*<.001, ***p*<.01, **p*<.05 | | | | | | | | |  |
|  |  | | | | | | | | |

**Supplementary Material S4a. Implicit theories of beauty stimuli in Studies 2, 3, and 4**


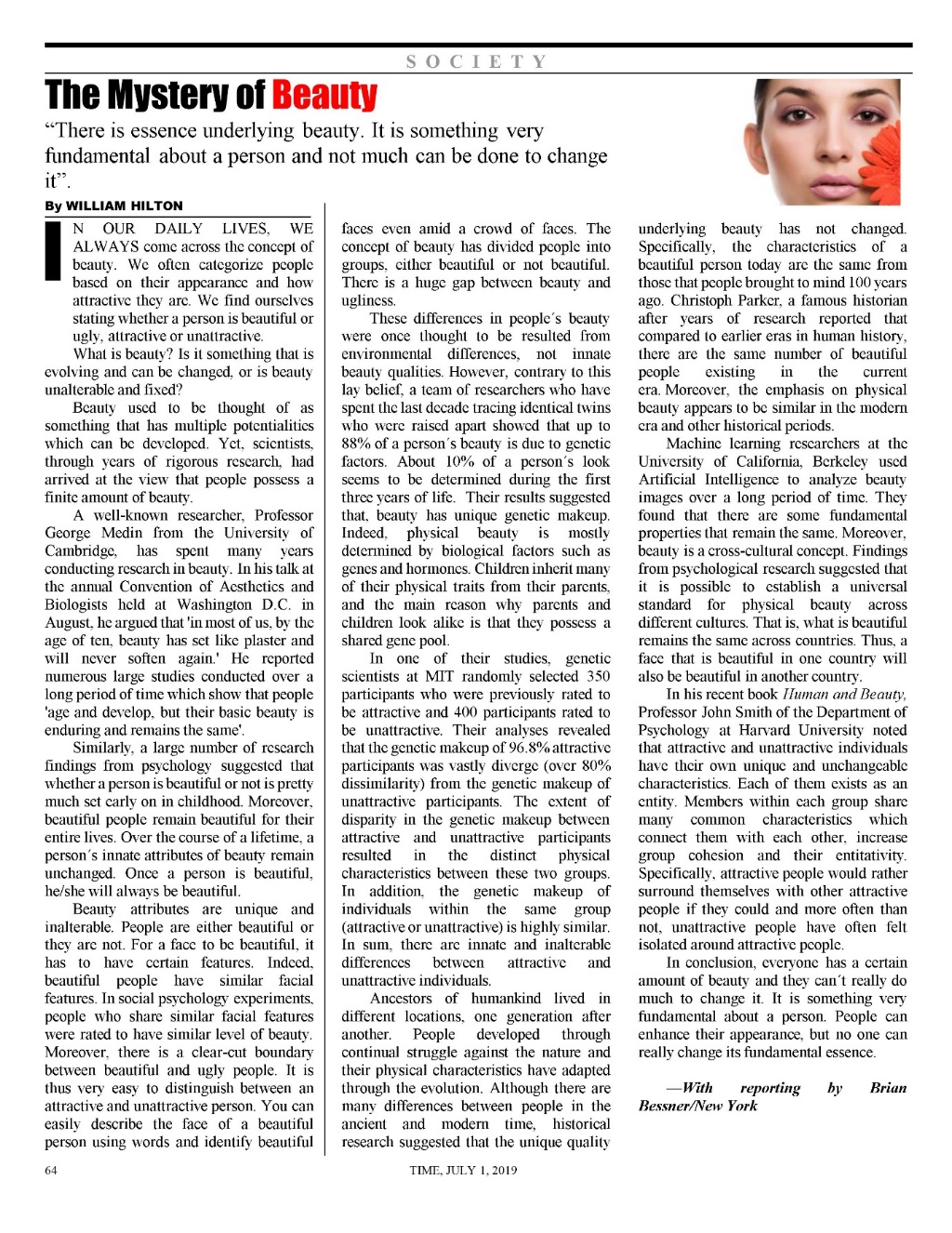


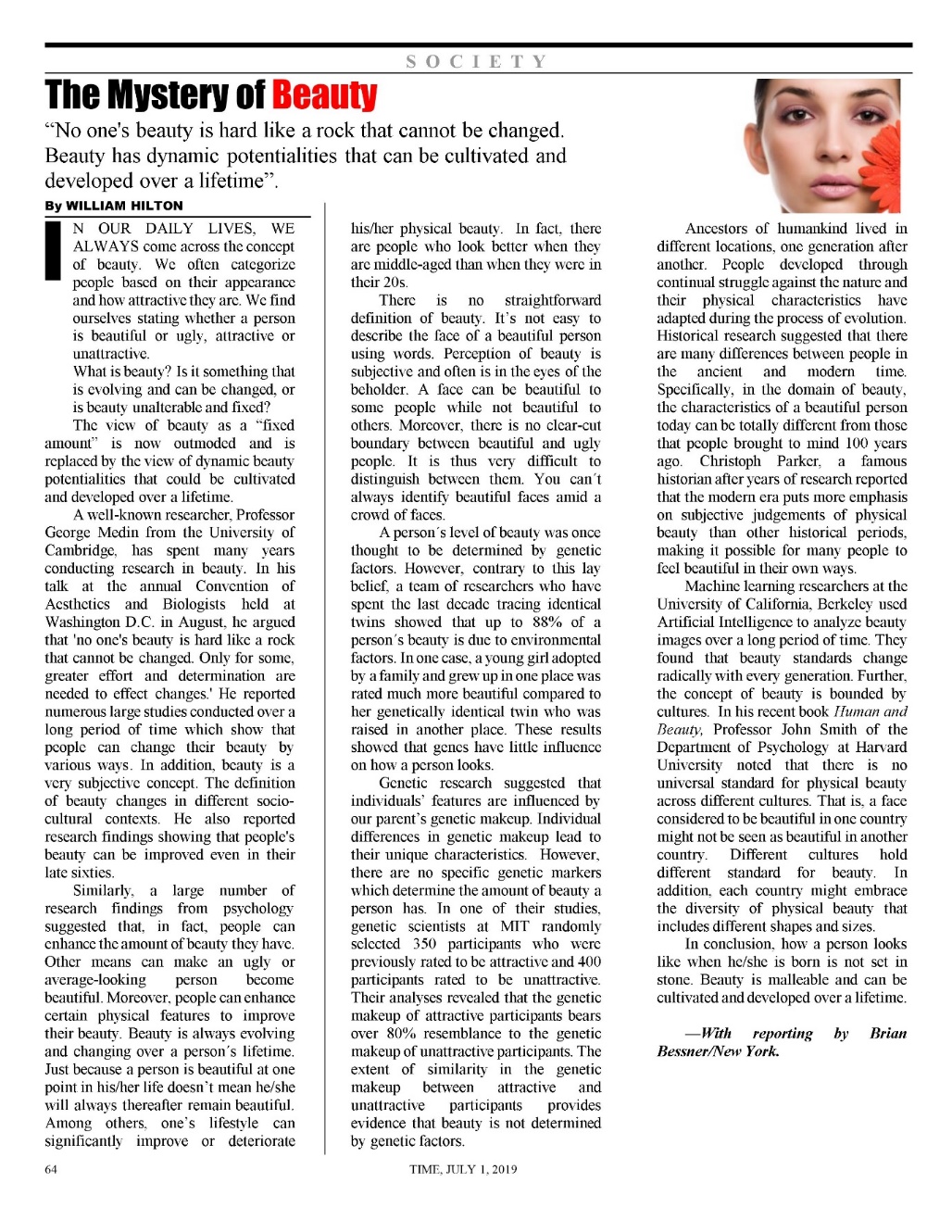


**Supplementary Material S4b. Implicit theories of beauty stimuli in Studies 6 and 7, and Supplementary Studies A, B, and C**

**
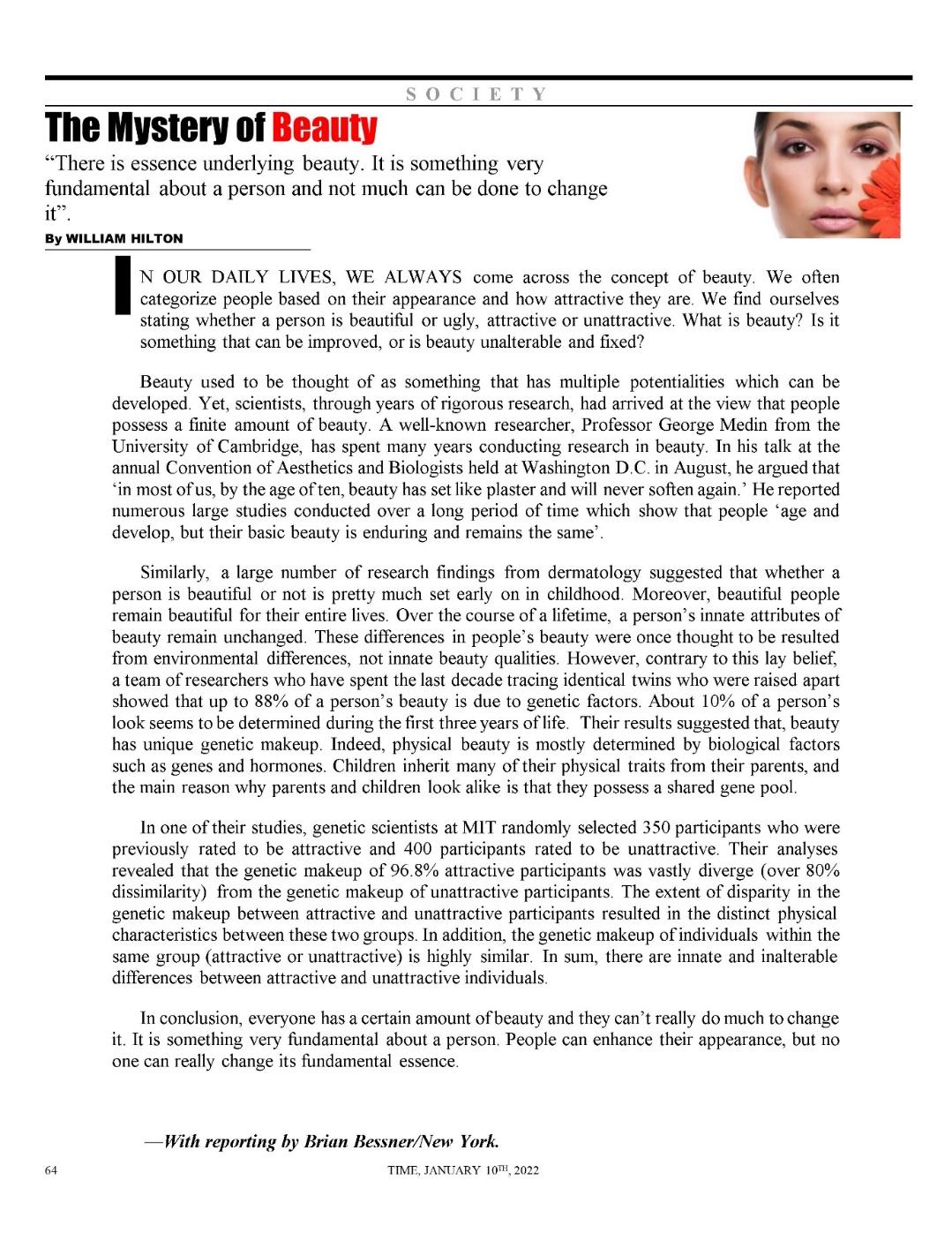
**

**
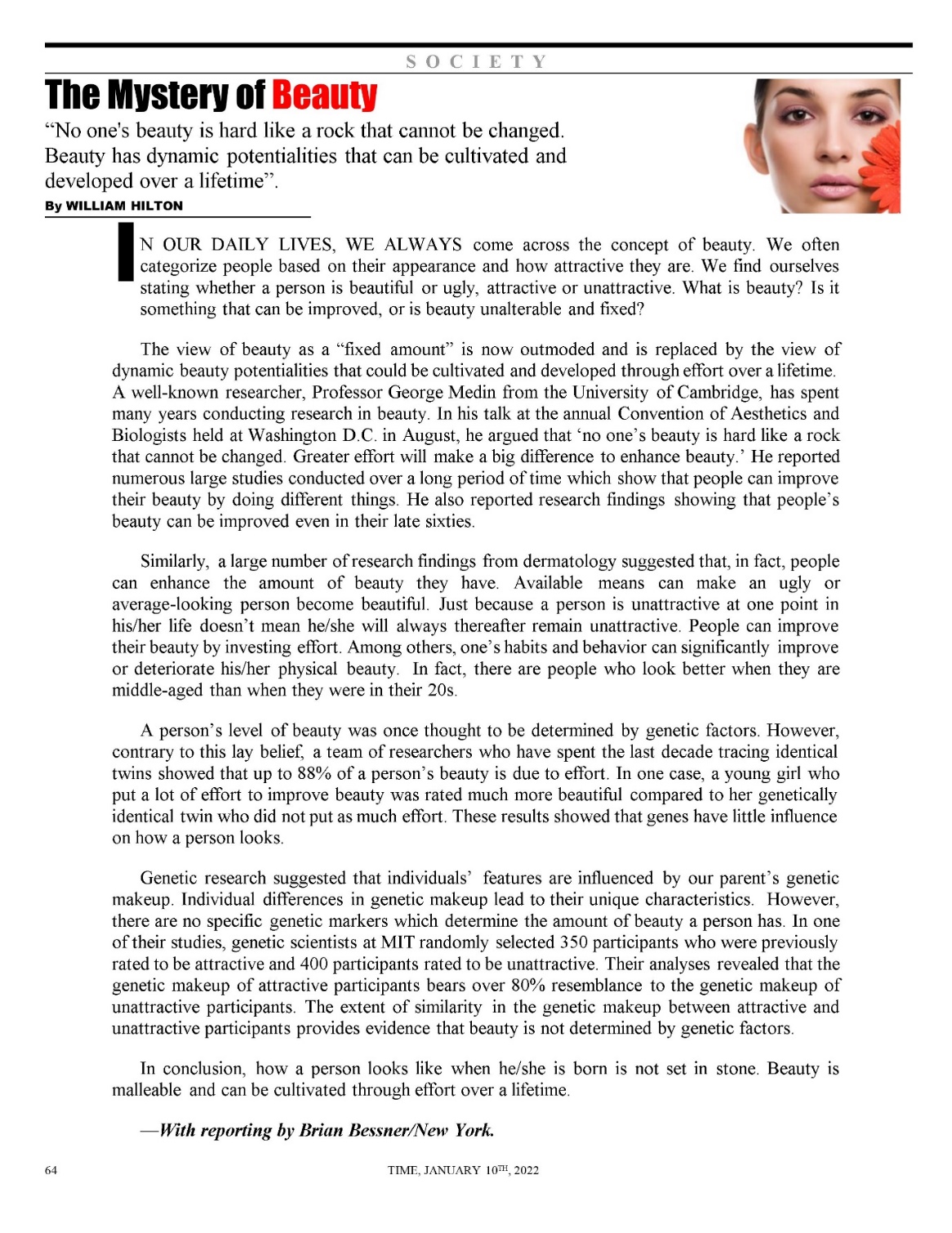
**

**Supplementary Material S5a. Pre-test for implicit theories of beauty manipulation in**

**Studies 2, 3, and 4**

To test the effectiveness of this implicit theory manipulation, a separate pre-test was conducted among 125 participants on Amazon Mechanical Turk (63.2% females, *M*_age_ = 34.38, SD = 9.34). Following the reading comprehension task, participants indicated their belief in the fixedness of beauty using the same three items as in study 1 (α = .88). Further, participants indicated how difficult it was to understand the article (1 = not at all difficult; 7 = extremely difficult) and the extent to which the article was believable (1 = not at all believable; 7 = extremely believable). After removing 27 participants who failed to provide a meaningful summary of the article, the final sample of the pre-test included 98 participants. An independent t-test confirmed that our implicit theories of beauty manipulation was successful. Perceived fixedness of beauty was higher in the entity theory than in the incremental theory conditions (*M*_entity_ = 4.58, SD = 1.49 vs. *M*_incremental_ = 3.35, SD = 1.50; *t*(96) = 4.05, *p* < .001, *d* = .82). Additional t-tests showed that both articles were considered easy to understand (*M*_entity_ = 2.86, SD = 1.65 vs. *M*_incremental_ = 2.44, SD = 1.41; *t*(96) = 1.36, *p* = .18, *d* = .27) and believable (*M*_entity_ = 4.50, SD = 1.59 vs. *M*_incremental_ = 4.69, SD = 1.43; *t*(96) = -.61, *p* = .54, *d* = .12).

**Supplementary Material S5b. Pre-test for implicit theories of beauty manipulation**

**in Studies 6 and 7, and Supplementary Studies A, B, and C**

Two hundred and one participants from Prolific (71.1% females; Mage = 33.61, SD = 12.78) were randomly assigned to one of the two articles. After reading the article, participants wrote down the theme of the article and provide one example from their experience to support the theme of the article. Participants then indicated their belief in the fixedness of beauty using the same three items as the pre-test above. An independent t-test showed that perceived fixedness of beauty was higher in the entity theory than in the incremental theory conditions (*M*_entity_ = 4.52, SD = 1.49 vs. *M*_incremental_ = 3.09, SD = 1.46; *t*(199) = -6.85, *p* < .001, *d* = .97).

**Supplementary Material S6. Rationale for including control variables in Study 2**

Several control variables were considered. First, participants’ perceptions of the effectiveness of different beautification methods that they are aware of in daily lives may correlate with the perceived malleability of beauty. Thus, we provided a list of methods participants may use to beautify themselves (e.g., make-up, skincare products, healthy diet, cosmetic surgery, etc.) and asked participants to rate each method in terms of how effective they are in taking care of their appearance (1=not effective at all, 7=very effective).

Second, participants’ perception of their own beauty might affect how implicit theories influence them (Hong et al., 1999). Following Hong et al. (1999), we showed participants three pairs of statements in which one statement illustrating a high level of affirmation for their beauty was pitted against another statement illustrating a low level of such affirmation (e.g., “I usually think I am attractive” vs. “I wonder if I am attractive”; all items in Supplementary Material S7). Participants indicated the extent to which their chosen statement was true for them (1=a little true, 5=extremely true). Responses to this measure were then recoded as a 10-point scale, ranging from low to high self-beauty perception (α=.92).

Third, participants’ perceived importance of being beautiful was considered. To the extent that beauty is considered important to participants, their belief in its improvability and malleability may play a role in their behavior. The perceived importance of being beautiful was measured with six items adapted from the literature (Crocker et al., 2003) (e.g., “Being good looking is very important to me”, 1=strongly disagree, 7=strongly agree; α=.82; see Supplementary Material S7 for all items).

Fourth, we measured trait risk-taking using the same measure as in Study 1 (α=.87). Finally, we included the Positive and Negative Affect Schedule (PANAS) Short Form (I-PANAS-SF; Thompson, 2007; Watson et al., 1988) to measure mood. We measured mood again in Studies 3 and 4 and did not find any significant difference in mood between entity and incremental theory conditions. Further, the effect of implicit theory of beauty on risk-taking remained significant controlling for mood.

**Supplementary Material S7. Measures of control variables in Study 2**

**Perceived effectiveness of beautification means:**

Please rate the following methods in terms of how effective they are in taking care of your beauty: Applying makeup; Using skincare products; Healthy diet; Exercise; Drinking water; Getting enough sleep; Cosmetic surgery; Plastic surgery

*(1 = not effective at all, 7 = very effective)*

**Perception of own beauty:**

Three pairs of statements in which one statement illustrating a high level of affirmation for their beauty was pitted against another statement illustrating a low level of such affirmation: “I usually think I am attractive” vs. “I wonder if I am attractive”; “I am pretty confident that I am attractive” vs. “I am not very confident that I am attractive”; and “I am sure that I look attractive” vs. “I am not sure that I look attractive”.

**Perceived importance of being beautiful:**

Looking beautiful can significantly change my future

Being good looking is very important to me

Attractiveness is important for my success in life

When I think I look attractive/beautiful, I feel good about myself

How I feel about myself is influenced by how attractive/beautiful I think

My sense of self-worth suffers whenever I think I do not look good

*(1 = strongly disagree, 7 = strongly agree)*

**Trait risk-taking** (Weber et al., 2002)

Betting a day’s income at the horse races

Investing 10% of your annual income in a moderate growth mutual fund

Betting a day’s income at a high stake poker game

Investing 5% of your annual income in a very speculative stock

Betting a day’s income on the outcome of a sporting event (e.g., baseball, soccer, or football)

Investing 5% of your annual income in a conservative stock

Investing 10% of your annual income in government bonds (treasury bills)

Gambling a week’s income at a casino

*(1 = not likely at all; 7 = very likely)*

**Mood:**

Please indicate how you feel at this moment: upset, hostile, alert, ashamed, inspired, nervous, determined, attentive, active, afraid *(1 = not at all, 7 = very much).*

**Supplementary Material S8. Results on control variables in Study 2**

*Control variables.* Independent t-tests showed no significant differences between entity and incremental beauty theorists in perceived effectiveness of various beautification methods (see Table S2 for the statistics). To understand whether any of these variables influenced the effect of implicit theories on the risk-taking measure, an ANCOVA was conducted with the number of pumps on unexploded balloons as the dependent variable and implicit theories as the independent variable. These control variables were treated as covariates (see Correlation matrix in Table S3). The effect of implicit theories of beauty on the number of pumps on unexploded balloons remained significant (*F*(1, 335)=11.44, *p*<.001, *η^2^*=.03).

*Table S2. Results on control variables in Study 2*

| **Control variables** | **Incremental condition**  **M *(SD)*** | **Entity condition**  **M *(SD)*** | **Independent t-tests statistics** |
| --- | --- | --- | --- |
| Applying makeup | 4.29 *(2.06)* | 4.20 *(1.98)* | *t*(349) = .39, *p* = .70, *d* = .04 |
| Using skincare products | 5.45 *(1.61)* | 5.21 *(1.59)* | *t*(349) = 1.40, *p* = .16, *d* = .15 |
| Healthy diet | 6.09 *(1.11)* | 6.10 *(1.07)* | *t*(349) = -.04, *p* = .97, *d* = .01 |
| Exercise | 6.04 *(1.31)* | 5.92 *(1.29)* | *t*(349) = .87, *p* = .38, *d* = .09 |
| Drinking water | 6.26 *(1.12)* | 6.18 *(1.07)* | *t*(349) = .63, *p* = .53, *d* = .07 |
| Getting enough sleep | 6.18 *(1.26)* | 6.11 *(1.10)* | *t*(349) = .53, *p* = .60, *d* = .06 |
| Cosmetic surgery | 3.54 *(1.88)* | 3.64 *(2.07)* | *t*(349) = -.47, *p* = .64, *d* = .05 |
| Plastic surgery | 3.37 *(1.86)* | 3.30 *(1.97)* | *t*(349) = .31, *p* = .76, *d* = .03 |
| Perception of participants’ own beauty | 6.02 *(2.89)* | 5.95 *(2.98)* | *t*(349) = .24, *p* = .81, *d* = .03 |
| Perceived importance of beauty | 4.93 *(1.10)* | 4.87 *(1.13)* | *t*(349) = .44, *p* =.66, *d* = .05 |
| Trait risk-attitude | 3.19 *(1.28)* | 3.23 *(1.39)* | *t*(349) = -.26, *p* = .80, *d* = .03 |
| Positive mood | 4.44 *(1.12)* | 4.27 *(1.13)* | *t*(348) = 1.39, *p* = .17, *d* = .15 |
| Negative mood | 2.27 *(1.25)* | 2.35 *(1.33)* | *t*(348) = -.62, *p* = .54, *d* = .07 |

*Table S3. Correlation matrix in Study 2*


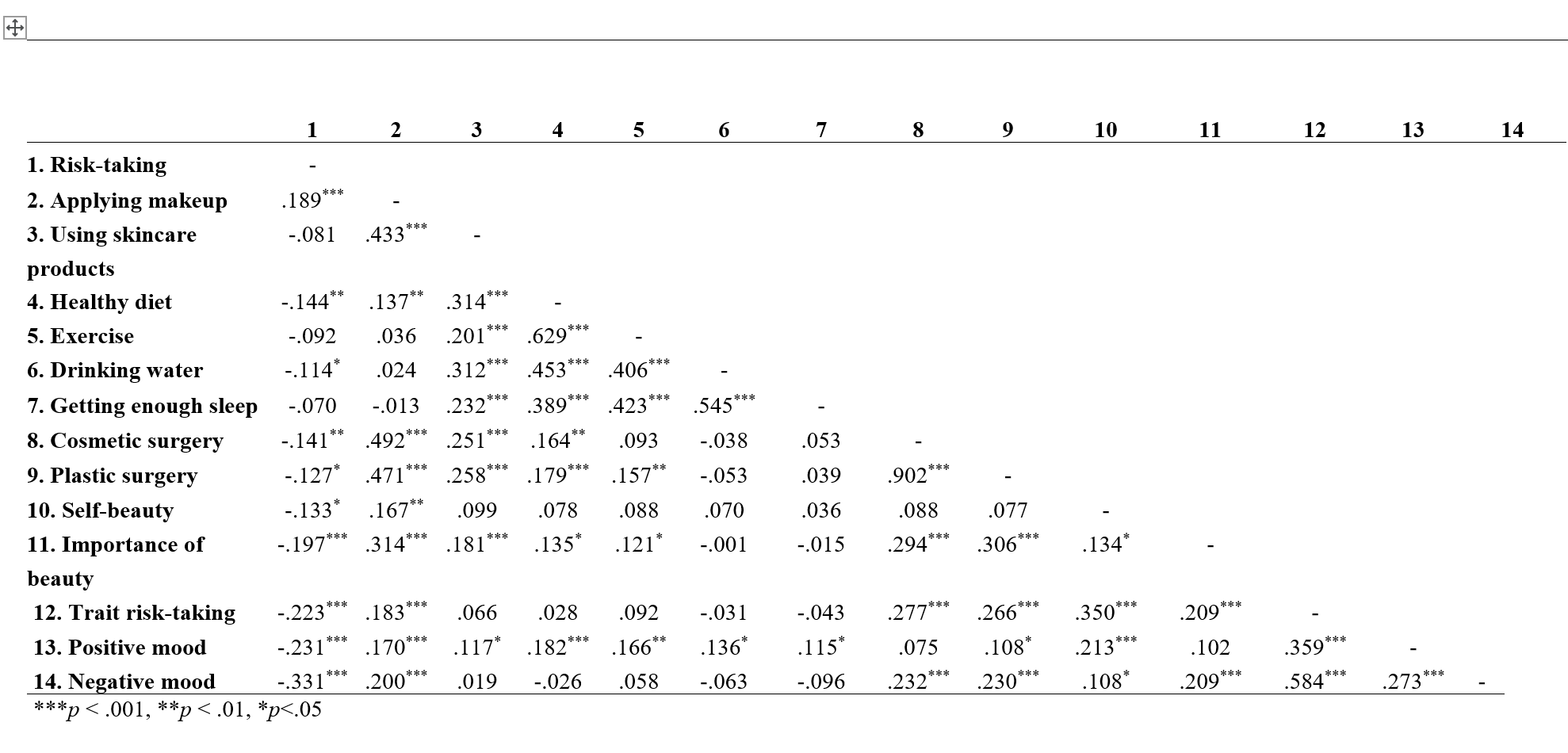


**Supplementary Material S9. Implicit theories manipulation check results across studies**

**Study 3:**

Results from an ANOVA on perceived fixedness of beauty revealed a significant effect of implicit theories (*F*(2, 458) = 7.79, *p* < .001, *η^2^* = .03). Perceived fixedness of beauty was lower in the incremental condition than the entity condition (*M*_incremental_ = 3.94, SD = 1.39 vs. *M*_entity_ = 4.60, SD = 1.43, *p* < .001, *d* = .47) and the control condition (*M*_incremental_ = 3.94, SD = 1.39 vs. *M*_control_ = 4.46, SD = 1.44, *p* = .002, *d* = .37).

**Study 4:**

We included a manipulation check for implicit theories of intelligence, with three items adopted from Dweck et al. (1995): “You have a certain amount of intelligence, and you cannot do much to change it”, “Your intelligence is something about you that you cannot change very much”, and “You can learn new things, but you cannot really change your basic intelligence” (1 = strongly disagree; 7 = strongly agree; α = .93).

A 2 (Implicit theories: entity vs. incremental) ANOVA on the perceived fixedness of beauty showed a significant effect, such that entity (vs. incremental) theorists perceived greater fixedness of beauty (*M*_entity beauty_ = 4.25, SD = 1.52 vs. *M*_incremental beauty_ = 3.78, SD = 1.48; *F*(1, 208) = 5.15, *p* = .02, *η^2^* = .02). Similarly, a 2 (Implicit theories: entity vs. incremental) ANOVA on the perceived fixedness of intelligence revealed a significant effect; entity theorists indicated greater fixedness of intelligence than incremental theorists (*M*_entity intelligence_ = 4.21, SD = 1.79 vs. *M*_incremental intelligence_ = 2.47, SD = 1.41; *F*(1, 215) = 63.57, *p* < .001, *η^2^* = .23).

**Study 6:**

An independent t-test confirmed that our implicit theories of beauty manipulation was successful. Perceived fixedness of beauty was higher in the entity theory than in the incremental theory condition (*M*_entity_ = 4.35, SD = 1.62 vs. *M*_incremental_ = 3.18, SD = 1.58; *t*(200) = -5.20 *p* < .001, *d* = .73).

**Study 7:**

A 2 (Implicit theories of beauty: entity vs. incremental) × 2 (Beauty is broad belief: strong vs. weak) ANOVA on the perceived fixedness of beauty revealed a significant effect of implicit theories of beauty (*M*_entity_ = 4.31, SD = 1.80 vs. *M*_incremental_ = 3.24, SD = 1.51; *F*(1, 389) = 41.26, *p* < .001, *η^2^* =.10). The main effect of beauty is broad belief was not significant (*F*(1, 389) = .91, *p* = .34, *η^2^* =.002), and the interaction effect of theory and belief was not significant (*F*(1, 389) = .70, *p* = .40, *η^2^* =.002).

**Supplementary Study A:**

An independent t-test show that participants in the entity (vs. incremental) theory condition indicated higher perceived fixedness of beauty (*M*_entity_ = 4.44, SD = 1.87 vs. *M*_incremental_ = 3.15, SD = 1.38, *t*(202) = -5.62, *p* < .001, *d* = .79).

**Supplementary Study B:**

An independent t-test confirmed that our implicit theories of beauty manipulation was successful. Perceived fixedness of beauty was higher in the entity theory than in the incremental theory condition (*M*_entity_ = 4.29, SD = 1.79 vs. *M*_incremental_ = 3.01, SD = 1.42; *t*(196) = -5.57, *p* < .001, *d* = .79).

**Supplementary Study C:**

A 2 (Implicit theories of beauty: entity vs. incremental) × 2 (Optimism vs. control) ANOVA on the perceived fixedness of beauty revealed a significant main effect of implicit theories (*M*_entity_ = 4.61, SD = 1.67 vs. *M*_incremental_ = 3.59, SD = 1.29; *F*(1, 393) = 46.91, *p* < .001, *η^2^* = .11) and a non-significant main effect of optimism (*F*(1, 393) = .03, *p* = .86, *η^2^* < .001). The interaction effect of theory and optimism was significant (*F*(1, 393) = 4.11, *p* = .04, *η^2^* =.01). Simple effects analyses further showed that entity (vs. incremental) theorists indicated higher fixedness of beauty in both control (*M*_entity/control_ = 4.78, SD = 1.69 vs. *M*_incremental/control_ = 3.45, SD = 1.27, *F*(1, 393) = 39.39, *p* < .001, *η^2^* =.09) and optimism condition (*M*_entity/optimism_ = 4.45, SD = 1.65 vs. *M*_incremental/optimism_ = 3.73, SD = 1.30, *F*(1, 393) = 11.63, *p* < .001, *η^2^* =.03).

**Supplementary Materials S10. Supplementary Study A**

In Supplementary Study A, we aimed to replicate the effect observed in Study 2; that is, an incremental (vs. entity) theory of beauty will lead to greater risk-taking.

**Method**

*Participants and Design.* Two hundred and four respondents from Prolific (57.8% females, *M*_age_=42.75, SD=13.22) were randomly assigned to one of the two conditions in a single-factor (Implicit theories of beauty: entity vs. incremental) between-subjects design.

*Procedure.* We manipulated implicit theory in the same way as Study 2, although the article is a shorter version (see Supplementary Materials 4b, pretest in Supplementary Materials 5b). We then measured risk-taking with four items (adopted from White et al. [2024]). Participants were asked: “If you have the opportunity, how likely are you to engage in the following behaviors within the next month? (1) Hiking a potentially unsafe trail or going down a potentially dangerous ski slope; (2) Investing some of your income in a speculative stock or in a new business venture; (3) Asking your boss for a promotion or leaving your current well-paying job to start a new career you enjoy more; and (4) Disagreeing with a close friend or family member about a major issue, or moving to a new city far away from your friends and extended family (1=extremely unlikely, 7=extremely likely; α=.62).

As a manipulation check, participants indicated their belief in the fixedness of beauty using the same three items as in Study 1 (1=strongly disagree, 7=strongly agree; α=.94). Lastly, participants indicated their demographic information (i.e., age, gender, and socioeconomic status).

**Results and Discussion**

An independent t-test with implicit theories of beauty as the independent variable and risk-taking as the dependent variable revealed that incremental theorists indicated greater risk-taking compared to entity theorists (*M*_incremental_=2.90, SD=1.22 vs. *M*_entity_=2.56, SD=1.11, *t*(202)=2.09, *p*=.038, *d*=.29). This study provided more evidence that an incremental theory of beauty lead to greater risk-taking compared to an entity theory of beauty, replicating the effect found in Study 2.

**Supplementary Material S11. Risk-taking measure in Study 3** (Hsee & Weber, 1999; Levav & Argo, 2010**)**

In this task, you will be making a choice between two options.

receive $400 for sure flip a coin; receive $2000 if Head or $0 if Tail

receive $600 for sure flip a coin; receive $2000 if Head or $0 if Tail

receive $800 for sure flip a coin; receive $2000 if Head or $0 if Tail

receive $1000 for sure flip a coin; receive $2000 if Head or $0 if Tail

receive $1200 for sure flip a coin; receive $2000 if Head or $0 if Tail

receive $1400 for sure flip a coin; receive $2000 if Head or $0 if Tail

receive $1600 for sure flip a coin; receive $2000 if Head or $0 if Tail

receive $20 for sure flip a coin; receive $100 if Head or $0 if Tail

receive $30 for sure flip a coin; receive $100 if Head or $0 if Tail

receive $40 for sure flip a coin; receive $100 if Head or $0 if Tail

receive $50 for sure flip a coin; receive $100 if Head or $0 if Tail

receive $60 for sure flip a coin; receive $100 if Head or $0 if Tail

receive $70 for sure flip a coin; receive $100 if Head or $0 if Tail

receive $80 for sure flip a coin; receive $100 if Head or $0 if Tail

**Supplementary Material S12. Results with control variables in Study 3**

*Control variables.* A multivariate analysis of variance (MANOVA) showed no significant differences of the condition (entity vs. incremental vs. control) in perceptions of participants’ own beauty (F(2, 543) = .32, *p* = .73, *η^2^* = .001), perceived importance of being beautiful (F(2, 543) = .84, *p* =.43, *η^2^* = .003), trait risk-attitude (F(2, 543) = .86, *p* = .42, *η^2^* = .003), positive mood (F(2, 543) = .64, *p* = .53, *η^2^* = .002), and negative mood (F(2, 543) = .64, *p* = .53, *η^2^* = .002). Further post-hoc Tukey’s HSD tests showed that the differences in these control variables between entity (vs. incremental vs. control) conditions were not significant (*p*_s_ > .4).

To understand whether any of these variables influenced the effect of implicit theories on the risk-taking measure, an ANCOVA was conducted with the number of riskier choices as the dependent variable and implicit theories as the independent variable. These control variables were treated as covariates (see Correlation matrix in Table S4 below). The effect of implicit theories of beauty on the number of riskier choices remained significant (*F*(2, 538) = 9.44, *p* < .001, *η^2^* = .03).

| *Table S4. Correlation matrix in Study 3* | | | | | | |  |
| --- | --- | --- | --- | --- | --- | --- | --- |
|  | **1** | **2** | **3** | **4** | **5** | **6** | |
| **1. Self-beauty** | - |  |  |  |  |  | |
| **2. Importance of beauty** | .222^***^ | - |  |  |  |  | |
| **3. Trait risk-taking** | .373^***^ | .393^***^ | - |  |  |  | |
| **4. Positive mood** | .317^***^ | .251^***^ | .341^***^ | - |  |  | |
| **5. Negative mood** | .065 | .269^***^ | .457^***^ | .182^***^ | - |  | |
| **6. Risk-taking** | .126^**^ | .104^*^ | .296^***^ | .109^*^ | .203^***^ | - | |

****p*<.001, **p*<.05

**Supplementary Materials S13. Results with control variables in Study 4**

*Control variables.* A multivariate analysis of variance (MANOVA) showed no interaction effect of implicit theories and domains on perceptions of participants’ own beauty/intelligence (F(1, 423) = .01, *p* = .92, *η^2^* = .006), perceived importance of being beautiful/intelligent (F(1, 423) = .81, *p* =.37, *η^2^* = .002), trait risk-attitude (F(1, 423) = 3.30, *p* = .07, *η^2^* = .008), positive mood (F(1, 423) = 1.64, *p* = .20, *η^2^* = .004), and negative mood (F(1, 423) = 1.77, *p* = .18, *η^2^* = .004).To understand whether any of these variables influenced the effect of implicit theories on the risk-taking measure, an ANCOVA was conducted with the preference for the riskier lottery options as the dependent variable and implicit theories as the independent variable. These control variables were treated as covariates. The effect of implicit theories of beauty on risk-taking remained significant (*F*(1, 418) = 4.74, *p* = .03, *η^2^* = .01).

**Supplementary Materials S14. Supplementary Study B**

**Method**

This study was pre-registered (<https://aspredicted.org/s2ks-r267.pdf>). Two hundred participants from Prolific (67% females, *M*_age_=36.92, SD=13.04) were randomly assigned to one of the two conditions in a one-factor (Implicit theories of beauty: entity vs. incremental) between-subjects design.

We manipulated implicit theories of beauty in a similar way as in the previous studies. Following the implicit theories of beauty manipulation, we asked participants to share their opinion about another Prolific study, in which they will be asked to complete a balloon pumping task. We explained the rules of the BART task, the same task that we used in Study 2. Further, we told participants that: “Imagine that in this study, your final score obtained in this task will be compared with other participants”. Participants then completed a real practice BART task with 10 trials, followed by the optimism measure: “How likely do you think you will get the highest point in the balloon pumping task?” (1=very unlikely, 7=very likely). We then included a manipulation check for implicit theories of beauty with the same three items (α=.95) as in previous studies.

**Results and Discussion**

As preregistered, we excluded two participants who failed to provide meaningful summary of the beauty articles, leaving a final sample of 198 participants. Without exclusions the patterns of results are the same.

*Optimism.* An independent t-test showed that beauty incremental theorists were more optimistic about their future performance compared to entity theorists (*M*_entity_=3.46, SD=1.33 vs. *M*_incremental_=3.96, SD=1.15; *t*(196)=2.83, *p*=.005, *d*=.40).

This study confirms a core assumption for our conceptualization—an incremental (vs. entity) beauty belief enhances optimism. Building on results of this study, in the Supplementary Study C, we aim to examine the role of optimism as the underlying mechanism by employing a process-by-moderation approach.

**Supplementary Materials S15. Supplementary Study C**

In this study (pre-registration: <https://aspredicted.org/px3k-5ghm.pdf>), we directly manipulated optimism to further demonstrate that optimism underlies the effect of implicit theories of beauty on risk-taking. If heightened optimism indeed increases risk-taking, then increasing the level of optimism should lead to higher risk-taking among beauty entity theorists as well.

**Method**

*Participants and Design.* A total of 400 participants from Prolific (58.5% females, *M*_age_=33.63, SD=12.30) were randomly assigned to one of the four cells in a 2 (Implicit theories of beauty: entity vs. incremental) × 2 (Optimism vs. control) between-subjects design.

*Procedure.* We manipulated implicit theories using the same stimuli as in previous studies. After this manipulation, we manipulated optimism by having participants complete a writing task. In the optimism condition, participants were instructed to recall *two* situations where they held positive, optimistic expectations before the outcomes became known to them (adapted from Chan et al., 2013). In the control condition, participants were asked to write about what they did yesterday.

To test the effectiveness of the optimism manipulation, we conducted a separate pre-test among 100 participants on Prolific (55% females, *M*_age_ = 35, SD = 13.90). Participants were randomly assigned to one of the two conditions (optimism vs. control). In the optimism condition, participants were instructed to recall *two* situations where they held positive, optimistic expectations before the outcomes became known to them (adopted from Chan et al., 2013) and to write them down. In the control condition, participants were asked to write about what they did yesterday. Then, they indicated their agreement to four statements measuring optimism (adopted from Cheung et al., 2013): “Thinking about the events I wrote about (1) makes me feel ready to take on new challenges, (2) makes me feel optimistic about my future, (3) makes me feel like the sky is the limit, and (4) gives me a feeling of hope about my future (1=strongly disagree, 7=strongly agree). Results confirmed that participants in the optimistic condition indeed indicated a higher level of optimism than participants in the control condition (*M*_optimistic_ = 4.81, SD = 1.45 vs. *M*_control_ = 3.95, SD = 1.33; *t*(98) = 3.08, *p* = .003, *d* = .62).

In the main study, after the manipulation check of optimism was administered (Cheung et al., 2013), participants completed the BART task as in previous studies; they were informed that the participant who has the highest points will get a £20 bonus. Finally, we included implicit theories of beauty manipulation check and demographic information.

**Results**

*Optimism manipulation check*. A 2 (Implicit theories of beauty: entity vs. incremental) × 2 (Optimism vs. control) ANOVA on optimism measure showed a significant main effect of theory (*M*_entity_ = 4.21, SD = 1.55 vs. *M*_incremental_ = 4.55, SD=1.21, *F*(1, 396) = 6.47, *p* = .01, *η^2^* =.02), and a significant main effect of optimism (*M*_optimism_ = 4.60, SD = 1.47 vs. *M*_control_ = 4.17, SD = 1.33, *F*(1, 396) = 9.88, *p* = .002, *η^2^* = .02). The interaction effect of theory and optimism was not significant (*F*(1, 396) = .29, *p* = .59, *η^2^* = .001), however simple effects analysis showed that incremental (vs. entity) theorists were more optimistic in the control condition (*M*_incremental/control_ = 4.37, SD = 1.28 vs. *M*_entity/control_ = 3.94, SD = 1.34 , *F*(1, 396) = 4.76, *p* = .03, *η^2^* = .01) whereas there was no significant difference between entity and incremental theorists in the optimism condition (*M*_incremental/optimism_ = 4.74, SD = 1.21 vs. *M*_entity/optimism_= 4.46, SD = 1.69 , *F*(1, 396) = 2.00, *p* = .16, *η^2^* = .005). This result is in line with our theory, that an incremental (vs. entity) theory of beauty increased optimism.

*Risk-taking.* A 2 (Implicit theories of beauty: entity vs. incremental) × 2 (Optimism vs. control) ANOVA on the number of pumps on unexploded balloons revealed no significant main effects of theory (*F*(1, 396)=2.44, *p*=.12, *η^2^*=.006) and optimism (*F*(1, 396)=1.33, *p*=.25, *η^2^*=.003), but a significant interaction effect of theory and optimism (*F*(1, 396)=12.51, *p*<.001, *η^2^*=.03) (Figure S1). Simple effects analyses showed that incremental (vs. entity) theorists pumped more balloons in the control condition (*M*_incremental/control_=651.20, SD=183.23 vs. *M*_entity/control_=543.94, SD=219.30, *F*(1, 396)=13.04, *p*<.001, *η^2^*=03), replicating the effect we found in the previous studies. By contrast, in the optimism condition, the effect of beauty theory attenuated (*M*_incremental/optimism_=601.07, SD=243.21 vs. *M*_entity/optimism_=642.60, SD=190.32, *F*(1, 396)=1.94, *p*=.16, *η^2^*= .01). Moreover, entity theorists pumped more in the optimism (vs. control) condition (*M*_entity/optimism_=642.60, SD=190.32, vs. *M*_entity/control_=543.94, SD=219.30, *F*(1, 396)=10.48, *p*=.001, *η^2^*=.03). On the other hand, there was no significant difference in the number of pumps between optimism and control conditions among incremental theorists (*M*_incremental/optimism_=601.07, SD=243.21 vs. *M*_incremental/control_=651.20, SD=183.23, *F*(1, 396)=2.99, *p*=.09, *η^2^*=.01).


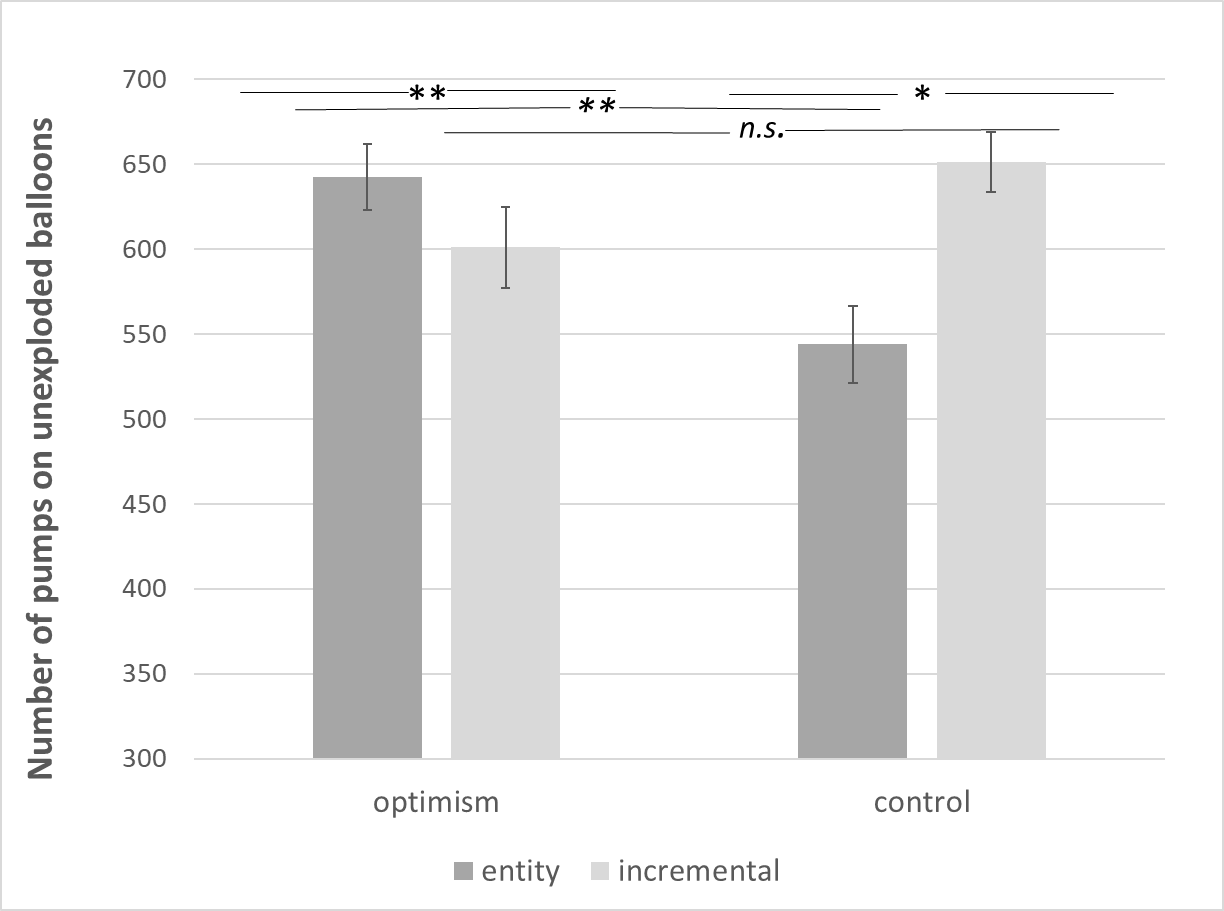


**Figure S1.** Supplementary Study C – Number of pumps on unexploded balloons as a function of implicit theories of beauty and optimism. Note: Error bars = +/- 1 SEs.

**Discussion**

This study demonstrated the moderating role of optimism on the effect of implicit theories of beauty on risk-taking, and thus demonstrated that incremental theorists’ greater risk-taking tendency was indeed driven by a heightened sense of optimism. In the control condition (i.e., no optimism manipulation), incremental theorists took more risks than entity theorists. When optimism was primed, entity theorists became as risk-seeking as incremental theorists. Supplementary Studies B and C provided evidence that optimism underlined the effect of implicit theories of beauty on risk-taking.

**Supplementary Material S16. Optimism measure in Study 6** (adapted from Cheung et al., 2013)

1. I am feeling ready to take on new challenges in many domains of life
2. I am feeling optimistic about my future in many domains of life
3. I am feeling like the sky is the limit in many domains of life
4. I am feeling hopeful about my future in many domains of life

*(1 = strongly disagree, 7 = strongly agree; α = .93).*

**Supplementary Material S17. Deviation from pre-registration in Study 7**

We note that there is a deviation from preregistration. We included a manipulation check of optimism “To what extent do you believe that beauty has a broad impact on various areas of life” (1=not at all, 7=very much). The results show that this manipulation did not work, however we suspected that the manipulation check was contaminated by the implicit theories of beauty manipulation check, which was measured right before. We thus instead confirmed the effectiveness of the manipulation by a pretest, as reported in the manuscript.

**Supplementary Material S18. Pretest for “beauty is broad” belief manipulation in Study 7**

**Method**

Two hundred and one participants from the U.S. on Prolific (51.7% females, Mage = 41.73, SD = 14.11) took part in the pretest.

We employed the same design as in the main study 7, in which participants were randomly assigned to one of the four conditions in a 2 (Implicit theories of beauty: entity vs. incremental) × 2 (“Beauty is broad” belief: strong vs. weak) between-subjects design. We used the same manipulation for beauty implicit theories as in previous studies. To manipulate “beauty is broad” belief, we employed a writing task. Specifically, participants read that “Some people believe that beauty is linked to many domains of life. That is, beauty has a broad impact on various areas”. Participants in the strong (vs. weak) belief condition were asked to provide 8 (vs. 2) examples to support this view.

Following the writing task, we measured “beauty is broad” belief with one item: “To what extent do you believe that beauty has a broad impact on various areas of life?” (1 = not at all, 7 = very much). Finally, we measured demographic variables including age, gender, and socioeconomic status.

**Results**

A 2 (Implicit theories of beauty: entity vs. incremental) × 2 (“Beauty is broad” belief: strong vs. weak) ANOVA on “beauty is broad” belief measure showed only a significant main effect of “beauty is broad” belief, with participants who provided 8 examples (vs. 2 examples) indicated greater score on this belief (*M*_8 examples_ = 6.12, SD = .85 vs. *M*_2 examples_ = 5.51, SD = 1.02; *F*(1, 197) = 21.17, *p* < .001, *η^2^* = .10). The main effect of implicit theories of beauty (*F*(1, 197) = .48, *p* = .49, *η^2^* = .002) and the interaction effect of implicit theories and beauty is broad belief (*F*(1, 197) = 2.48, *p* = .12, *η^2^* = .012) were not significant.

**Supplementary Materials S19. Major analyses on the role of gender in the impact of beauty implicit theories on risk-taking**

**Study 1:** We conducted a moderation analysis using Hayes’ Process (Hayes, 2017) model 1, with beauty implicit theories as the independent variable, risk-taking as the dependent variable, gender as the moderator, and the four cultural dimensions as covariates. Results showed a significant main effect of beauty implicit theories (*B*=.98, SE=.13, *t*=7.54, *p*<.001), a significant main effect of gender (*B*=-.30, SE=.10, *t*=-2.96, *p*=.003), and a significant interaction of beauty implicit theories and gender (*B*=-.24, SE=.08, *t*=-2.81, *p*=.005). For both men and women, there was a significant positive effect of beauty implicit theories on risk-taking (men: *B*=.74, SE=.06, *t*=12.86, *p*<.001; women: *B*=.51, SE=.06, *t*=7.95, *p*<.001).

**Study 2:** An ANOVA with beauty implicit theories and gender as independent variables and risk-taking as the dependent variable showed only a significant main effect of beauty implicit theories (*F*(1, 346) = 9.62, *p*=.002). The main effect of gender (*F*(1, 346)=.01, *p*=.91) and the interaction effect of beauty implicit theories and gender (*F*(1, 346)= .18, *p*=.68) were not significant.

**Study 3:** An ANOVA with beauty implicit theories (incremental vs. entity vs. control) and gender as factors, and risk-taking as the dependent variable showed a significant main effect of beauty implicit theories (*F*(1, 540) = 11.22, *p*<.001), a significant main effect of gender (*F*(1, 540)= 22.07, *p*<.001), and a significant interaction effect of beauty implicit theories and gender (*F*(2, 540)=3.01, *p*=.05). Simple-effects tests showed that men in the incremental theory condition took more risk compared to men in the entity condition (*p*<.001) and control condition (*p*<.001). Women in the incremental theory condition did not take more risks compared to entity (but marginally significant, *p*=.08) and control condition (*p*=.13).

**Study 4:** An ANOVA with beauty implicit theories, domain (beauty vs. intelligence), and gender as factors and risk-taking as the dependent variable showed only a significant main effect of domain (*F*(1, 417)=12.20, *p*<.001) and a significant interaction effect of implicit theories and domain (*F*(1, 417) = 7.57, *p*=.006). Other effects were not significant (*p*_s_ > .1).

**Study 5:** An Independent t-test showed that there is no difference in the D-score between men and women (*p*=.70), indicating that both men and women similarly hold a beauty-optimism association.

**Study 6**: We conducted Hayes’ Process model 8 with beauty implicit theories as the independent variable, risk-taking as the dependent variable, optimism as the mediator, and gender as the moderator. Results revealed that beauty implicit theories and gender did not interactively influence optimism (*B*=.27, SE=.41, *t*=.65, *p*=.52) and risk-taking (*B*=-.21, SE=.32, *t*=-.66, *p*=.51).

**Study 7**: An ANOVA with beauty implicit theories, beauty is broad belief, and gender as factors and risk-taking as the dependent variable showed a significant interaction effect of beauty implicit theories and beauty is broad belief (*F*(1, 380)=4.53, *p*=.03), and a significant interaction of beauty is broad belief and gender (*F*(3, 380)=3.24, *p*=.02). Other effects were not significant (*p*_s_>.05). The three-way interaction of beauty implicit theories, beauty is broad belief, and gender was not significant (*p*=.85). Simple-effect tests showed gender differences; for men when the belief that beauty is broad was strong (i.e., participants wrote 8 examples), incremental (vs. entity) theorists took more risks (*p*<.001). This effect was weaker for women (*p*=.059) and not significant for non-binary/third gender (*p*=.43). When the belief that beauty is broad was weakened, the effect disappeared for both men (*p*= .22) and women (*p*=.62).

**References**

[Chan](https://www.sciencedirect.com/science/article/abs/pii/S1057740812000617#!), E., [Sengupta](https://www.sciencedirect.com/science/article/abs/pii/S1057740812000617#!), J., & [Mukhopadhyay](https://www.sciencedirect.com/science/article/abs/pii/S1057740812000617#!), A. (2013). The antecedents of anticipatory purchase: Reconciling the two routes to optimism. *Journal of Consumer Psychology, 23*(1), 90-105.

Cheung, W. Y., Wildschut, T., Sedikides, C., Hepper, E. G., Arndt, J., & Vingerhoets, A. J. J. M. (2013). Back to the future: Nostalgia increases optimism. *Personality and Social Psychology Bulletin, 39*, 1484-1496.

Crocker, J., Luhtanen, R. K., Cooper, L. M., & Bouvrette, A. (2003). Contingencies of self-worth in college students: Theory and measurement. *Journal of Personality and Social Psychology, 85*(5), 894-908.

Dweck, C. S., Chiu, C., & Hong, Y. (1995). Implicit theories and their role in judgments and reactions: A world from two perspectives. *Psychological Inquiry, 6*(4), 267–285.

Hayes, A. F. (2017). Introduction to mediation, moderation, and conditional process analysis: A regression-based approach. Guilford publications.

Hong, Y., Chiu, C., Dweck, C. S., Lin, D. M. S., & Wan, W. (1999). Implicit theories, attributions, and coping: A meaning system approach. *Journal of Personality and Social Psychology, 77*(3), 588-599.

Hsee, C. K., & Weber, E. U. (1999). Cross-national differences in risk preference and lay predictions. *Journal of Behavioral Decision Making, 12*(2), 165-179.

Levav, J., & Argo, J. J. (2010). Physical contact and financial risk taking. *Psychological Science, 21*(6), 804-810.

Thompson, E. R. (2007). Development and validation of an internationally reliable short-form of the Positive and Negative Affect Schedule (PANAS). *Journal of Cross-Cultural Psychology, 38*, 227–242.

Triandis, H. C., & Gelfland, M. J. (1998). Converging measurement of horizontal and vertical individualism and collectivism. *Journal of Personality and Social Psychology, 74*, 118-128.

Watson, D., Clark, L. A., & Tellegen, A. (1988). Development and validation of brief measures of positive and negative affect: The PANAS scales. *Journal of Personality and Social Psychology, 54*(6), 1063-1070.

Weber, E. U., [Blais](https://onlinelibrary.wiley.com/action/doSearch?ContribAuthorStored=Blais%2C+Ann-Ren%C3%A9e) , A., & [Betz](https://onlinelibrary.wiley.com/action/doSearch?ContribAuthorStored=Betz%2C+Nancy+E), N. E. (2002). A domain-specific risk-attitude scale: Measuring risk perceptions and risk behaviors. *Behavioral Decision Making, 15*(4), 263-290.

White, C. J., Dean, C. M., & Laurin, K. (2024). Do reminders of God increase willingness to take risks?. *Journal of Experimental Social Psychology, 110*, 104539.
